# Harnessing Internet Search Data as a Potential Tool for Medical Diagnosis: Literature Review

**DOI:** 10.2196/63149

**Published:** 2025-02-11

**Authors:** Gregory J Downing, Lucas M Tramontozzi, Jackson Garcia, Emma Villanueva

**Affiliations:** 1 Innovation Horizons, Inc Washington, DC United States; 2 Department of Health Systems Administration School of Health Georgetown University Washington, DC United States

**Keywords:** health, informatics, internet search data, early diagnosis, web search, information technology, internet, machine learning, medical records, diagnosis, health care, self-diagnosis, detection, intervention, patient education, internet search, health-seeking behavior, artificial intelligence, AI

## Abstract

**Background:**

The integration of information technology into health care has created opportunities to address diagnostic challenges. Internet searches, representing a vast source of health-related data, hold promise for improving early disease detection. Studies suggest that patterns in search behavior can reveal symptoms before clinical diagnosis, offering potential for innovative diagnostic tools. Leveraging advancements in machine learning, researchers have explored linking search data with health records to enhance screening and outcomes. However, challenges like privacy, bias, and scalability remain critical to its widespread adoption.

**Objective:**

We aimed to explore the potential and challenges of using internet search data in medical diagnosis, with a specific focus on diseases and conditions such as cancer, cardiovascular disease, mental and behavioral health, neurodegenerative disorders, and nutritional and metabolic diseases. We examined ethical, technical, and policy considerations while assessing the current state of research, identifying gaps and limitations, and proposing future research directions to advance this emerging field.

**Methods:**

We conducted a comprehensive analysis of peer-reviewed literature and informational interviews with subject matter experts to examine the landscape of internet search data use in medical research. We searched for published peer-reviewed literature on the PubMed database between October and December 2023.

**Results:**

Systematic selection based on predefined criteria included 40 articles from the 2499 identified articles. The analysis revealed a nascent domain of internet search data research in medical diagnosis, marked by advancements in analytics and data integration. Despite challenges such as bias, privacy, and infrastructure limitations, emerging initiatives could reshape data collection and privacy safeguards.

**Conclusions:**

We identified signals correlating with diagnostic considerations in certain diseases and conditions, indicating the potential for such data to enhance clinical diagnostic capabilities. However, leveraging internet search data for improved early diagnosis and health care outcomes requires effectively addressing ethical, technical, and policy challenges. By fostering interdisciplinary collaboration, advancing infrastructure development, and prioritizing patient engagement and consent, researchers can unlock the transformative potential of internet search data in medical diagnosis to ultimately enhance patient care and advance health care practice and policy.

## Introduction

### Overview

The transition to an era in which IT plays a pivotal role in health care goes beyond an information engineering advancement to address a substantial medical necessity. Evidence is emerging that internet searches for medical information may help facilitate diagnoses of medical conditions. Machine learning models may predict the diagnosis of a condition more accurately than traditional diagnostic methods. Integration of internet search data with a patient’s medical records may provide an opportunity for enhanced screening to identify disease in its early stages. In response to nascent research in this area, the Gordon and Betty Moore Foundation is supporting an initiative to explore the potential to harness internet search data for medical diagnoses. This report reflects a component of a comprehensive research endeavor focused on addressing diagnostic delays before hospitalization, encompassing the time lapses preceding a patient’s arrival at a health care facility where their condition is conclusively diagnosed [1].

Through a review of the relevant peer-reviewed literature and informational interviews with subject matter experts, this report identifies key themes and insights to lay the groundwork for understanding the implications of leveraging internet search data that links with health research datasets resulting in innovative methodologies that empower health care professionals to make precise and timely diagnoses.

This work does not consider a patient’s search engine preference for finding health information, nor review the patterns, trends, or accuracy of patient self-diagnosis through internet searches. It does identify the current body of literature from researchers who leverage internet search data to link to other health research data about the individual patient to identify a diagnosis. Our objective is to explore the broader landscape of leveraging internet search data in health care and its potential for assisting clinicians with diagnoses and to elucidate promising avenues for researchers to enhance diagnostic capabilities through thoughtful application of internet search data. In doing so, we sought a nuanced understanding of the possibilities within the realm of health care diagnostics with a focus on leveraging search history data to benefit clinical care teams rather than investigating self-diagnosis pathways.

This paper sheds light on the research surrounding the use of consumer internet search data for early health concern detection without delving into the clinical validation of such findings. It focuses on the identification of potential diagnostic signals and patterns to inform predictive models and proactive health care interventions, while acknowledging the challenges involved in discerning such signals among the vast array of search queries. By leveraging insights from internet search data, health care professionals may more effectively identify early warning signs, enabling timelier interventions and improved patient outcomes.

### Background

#### Overview

Access to accurate medical diagnosis has been hindered by socioeconomic disparities, limited availability of specialized medical professionals, and lack of patient education, among other factors. Inequities in access to high-quality health care services exacerbate these challenges, leading to disparities in health outcomes. Missed or inaccurate diagnoses can lead to delayed or unnecessary treatments, risking worsening of conditions. The historical reliance on direct patient-physician interactions for diagnosis has failed to bridge these gaps. The emergence of the internet and digital data in the late 20th century began to alter this landscape. Eysenbach [2] highlighted the early potential of the internet in patient education, setting the stage for an ever-increasing reliance on online health information; however, questions remain regarding information accuracy, access, benefits, and privacy.

Internet searches represent one of the largest sources of health data. As of mid-2023, Google’s daily search volume was >8.5 billion queries [3]. Around 5% of Google searches are health related [4], and about 77% of persons with a new diagnosis use search engines [5]. A recent study showed that 15% of internet searches by individuals with a recent diagnosis involved symptoms of a disease before diagnosis [6], and 15% of all annual Google searches are new [7].

These and other data have prompted a series of research projects to address the feasibility and utility of using internet search data for seeking health services. Although the use of patient search data represents just one facet of technology being explored to help obtain new data about patient conditions [8], this paper focuses only on research that uses internet search data.

#### Population Health Research

In population health research, there are studies available to assist researchers in approaching the use of internet search data. These studies focus on population health rather than diagnostic search but remain valuable because they offer methodologies for leveraging internet search data that can benefit research. These studies also delve into how understanding the dynamics of vaccine hesitancy across social media is crucial in devising strategies to promote vaccine acceptance.

Forecasting vaccine hesitancy has become increasingly vital within public health initiatives, and internet search data and social media platforms are pivotal in comprehending the underlying dynamics of this hesitancy [9]. Leveraging data from search logs and social media platforms through machine learning and data analysis provides fresh perspectives on vaccine intentions and behaviors that aid policy makers and health care professionals in crafting strategies to tackle vaccine hesitancy.

The study titled “Accurate Measures of Vaccination and Concerns of Vaccine Holdouts from Web Search Logs” [10] showcases the potential of using search logs for insightful analysis that addresses health concerns of patients. By developing a vaccine intent classifier, researchers accurately detect user searches for COVID-19 vaccines that strongly correlate with the Centers for Disease Control and Prevention’s vaccination rates [10], enabling real-time estimation of vaccine intent rates across demographics and regions and revealing granular tendencies in vaccine-seeking behavior [10]. Machine learning identifies vaccine holdouts; their inclination toward using untrusted news sources; and specific concerns about vaccine requirements, development, and myths [10]. Understanding these concerns among demographic groups unveils variations in hesitancy, shedding light on those crucial moments when individuals transition from being vaccine holdouts to considering vaccination [10].

Similarly, the study on COVID-19 vaccine hesitancy and increased internet search queries for fertility side effects following emergency use authorization demonstrates the link between public concerns and vaccine uptake [11]. The surge in fertility-related queries after emergency use authorization, fueled by unfounded scientific claims propagated on social media, underscores the hesitancy regarding potential side effects that influenced vaccine acceptance rates [11], emphasizing the importance of addressing specific concerns highlighted by web searches to alleviate hesitancy and promote informed public decision-making.

Research involving empathic engagement with vaccine-hesitant individuals in private Facebook groups highlights the potential of social media platforms to provide a place for health education and discussions [12]. These moderated discussions positively influenced vaccination intentions and beliefs, representing a promising strategy for combating vaccine hesitancy [13].

Social media policies and interventions play an important role in mitigating vaccine misinformation. Policies implemented by platforms such as Facebook have reduced the reach of antivaccine content [14], and the systematic appraisal of current social media strategies and their alignment with evidence-based practices represent necessary first steps [15,16]. However, the primary focus of these studies involves public sentiment, intentions, and behavioral patterns and not the diagnosis of specific conditions. Leveraging internet search data and social media platforms provides insights into vaccine hesitancy that can drive evidence-based strategies to address hesitancy, promote informed decision-making, and contribute to the success of vaccination campaigns, potentially curbing the spread of vaccine misinformation during public health emergencies.

## Methods

### Literature Review

We conducted a literature search on the PubMed database from October 2 to October 30, 2023, using predefined keywords and Medical Subject Headings related to Google, Bing, Takeout, internet search, web search, search behavior, diagnosis, disease identification, and diagnostic accuracy. Multimedia Appendix 1 lists all the search terms.

Stringent inclusion criteria were applied to identify relevant studies for analysis according to the PRISMA (Preferred Reporting Items for Systematic Reviews and Meta-Analyses) guidelines (Multimedia Appendix 2). Inclusion was limited to studies that used internet search data from Google and Microsoft Bing, which account for >90% of all internet searches [3]. The selected studies’ primary focus was on individual diagnosis and health behavior to ensure a targeted exploration of search data applications in the context of personal health. Studies were required to integrate internet search data with other health research datasets to provide perspective on individual health outcomes and capture the synergistic potential of combining search data with other health-related information.

Exclusion criteria were established to maintain specificity and relevance to the research focus. Studies within the domain of broad population health research were excluded, as was research solely reliant on social media data. These inclusion and exclusion criteria were applied to pinpoint studies aligning with the project’s primary focus: leveraging patients’ internet search data for individual diagnosis and providing patients with information to aid in screening.

All articles retrieved from the initial PubMed database search were uploaded to Covidence (Veritas Health Innovation Ltd), where duplicates were removed, and the literature review process was conducted according to the predefined inclusion and exclusion criteria. To reduce errors and bias, the authors independently screened the papers’ titles and abstracts, and full texts of potentially eligible articles were examined for final inclusion. Throughout this process, the authors periodically compared findings, resolving any discrepancies through discussion and consensus to ensure thoroughness and accuracy in study selection.

Textbox 1 presents the inclusion and exclusion criteria used to screen publications on the basis of the title and abstracts. The exclusion criteria for this literature review were clearly defined to ensure the relevance and quality of the included studies.

**Inclusion and exclusion criteria.**

**Inclusion criteria**

- Article type: peer-reviewed journals
- Article focus: use of internet search data, both anonymized and fully identified
- Outcomes: the combined use of health research datasets and internet search histories to identify, predict, or confirm a clinical diagnosis
- Time: January 1, 2005, to October 30, 2023
- Language: English
- Geography: international

**Exclusion criteria**

- Article type: newspaper articles, opinions, and commentaries; articles unavailable in full-text format; duplicate studies
- Article focus: studies that did not verify a patient’s clinical diagnosis following analysis of internet search behavior; studies that focused solely on diagnoses at the population level, without specific individual-level data; articles primarily discussing moral, ethical, or privacy considerations related to the use of internet search data without providing analytical insights from the integration of search and clinical data
- Time: dated before January 1, 2005
- Language: non-English

### Informational Interviews

To complement the literature review, informational interviews were conducted with a convenience sample of experts, including researchers, patient advocates, ethicists, and professionals in the open data field, focusing on the use of internet search data in health research. These participants were identified through contacts provided by AcademyHealth. Web-based interviews were conducted over a 3-week period using a survey instrument (Multimedia Appendix 3) designed to explore topics such as the application of internet search data in health research, public acceptance and perceptions of its use, and privacy and security considerations. Thematic analysis was performed on the interview data to identify recurring insights and perspectives, which were then synthesized to complement the findings from the literature review and highlight critical considerations for advancing this field.

## Results

**Figure 1:**
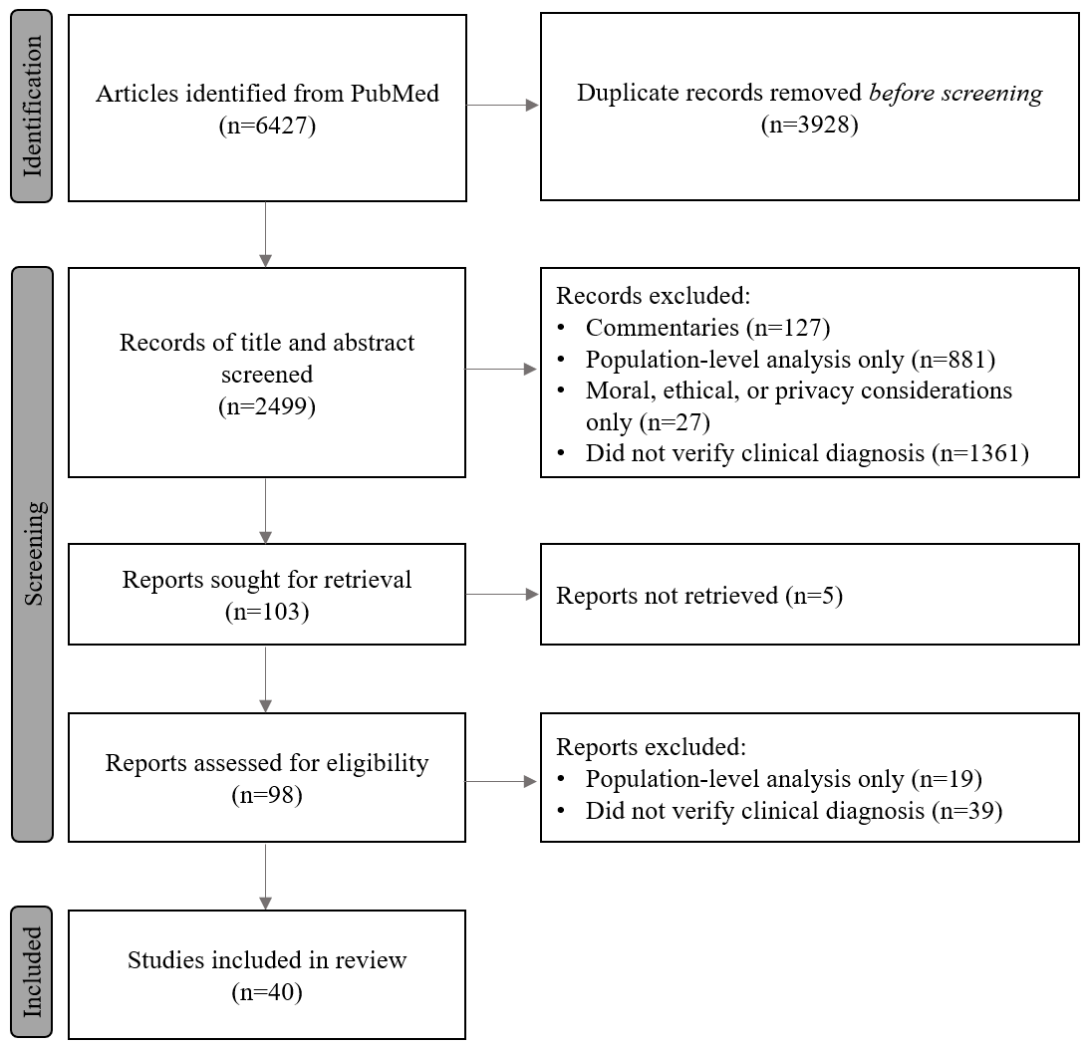
PRISMA (Preferred Reporting Items for Systematic Reviews and Meta-Analyses) flowchart for literature selection.

The search initially yielded 6427 articles, reports, and publications from the PubMed database. Figure 1 presents the PRISMA flowchart of the record selection process. Duplicates were removed from all articles identified across all searches totaling 3928 (ie, 61% of all results).

A total of 2499 peer-reviewed articles were selected for screening on the basis of title and abstract for inclusion and exclusion consideration following the focused criteria. A total of 2396 articles were excluded based on the following criteria: commentaries (n=127, 5.3%); focused only on population-level disease identification (n=881, 36.8%); focused predominantly on the moral, ethical, or privacy considerations for the use of internet search history while not presenting insights from the analysis of search and clinical data (n=27, 1%); or only investigated internet search data without confirming a diagnosis from an independent dataset or from the patient directly (n=1361, 56.8%).

Full-text reports were sought for the remaining 103 articles; however, 5 articles could not be retrieved. Of the 98 reports obtained, the authors read the full text and excluded 59% (n=55) of reports that focused primarily on a population-level analysis (n=19, 19%) or that did not confirm a diagnosis from an independent dataset or from the patient directly (n=39, 40%). This process resulted in the inclusion of 40 articles in this literature review (Multimedia Appendix 4 [6,8,17-54]).

Table 1 provides a categorized summary of the 40 peer-reviewed articles included in this literature review, organized by the disease or condition each study addresses. This categorization highlights the diverse applications of internet search data in diagnosing a range of health conditions, from cancer to mental and behavioral health, showcasing the breadth of this emerging research field. The table underscores the potential for leveraging search data across various medical domains to enhance diagnostic capabilities and early detection efforts.

A total of 16 interviews were conducted with participants from academic institutions (n=8, 50%), private-sector companies (n=5, 31%), and nonprofit organizations (n=3, 19%). Key findings highlighted several challenges and opportunities in the field, including difficulties in obtaining institutional review board (IRB) approval for studies involving internet search data, the importance of building trust between patients and researchers to encourage data sharing, and the need for streamlined processes to download and access search data. Participants also emphasized the lack of tools to effectively analyze unstructured data and build predictive models.

**Table 1:** Total articles by disease category (N=40).

Disease category	Articles, n (%)^a^
Cancer	13 (32)
Cardiovascular disease	2 (5)
Emergency care	6 (15)
Mental and behavioral health	16 (40)
Neurodegenerative diseases	5 (12)
Nutritional and metabolic diseases	4 (10)

^a^Some articles address multiple disease categories, causing the total in the table to exceed the total number of studies.

## Discussion

### Principal Findings

Within health and health services queries, 3 distinct categories demonstrate generalizability to population-based analyses and individual-specific applications on the basis of the search content and patterns. While the use of data from *aggregate, anonymized* queries is widespread, particularly in epidemiological studies at the population level, such use lacks value in diagnosing specific conditions for individual patients. These anonymized datasets, lacking individuals’ specific informed consent, fall under exempt research use. Consequently, this paper does not include detailed examinations of these studies [55,56].

The second and third categories of research applications using search queries are the focus of this examination. Both include individually consented patient data that may be linked with additional clinical datasets. In the United States, these studies fall under the Common Rule and Health Insurance Portability and Accountability Act (HIPAA) privacy rule [57].

The second category includes using a *search history to predict future search queries* strongly correlating with health conditions or disease outcomes. One application involves developing specialized queries associated with a condition, searching patients’ internet logs with that condition, and evaluating associated symptoms [17]. Researchers then build statistical classifiers to predict future appearances of landmark queries based on patterns in search logs [17]. Such signals show possibilities of predicting forthcoming diagnoses from subtle temporal signals in searchers’ queries [17]. This approach was used to establish patient searches of symptoms associated with pancreatic cancer before their clinical diagnosis [17].

The third category involves using users’ internet *search logs for which they granted consent for researchers* to access and possibly permitted linkage to their health data. Most applied research in this category has used retrospective analysis to correlate with features of clinical symptoms or diagnostic tests. In several cases, particularly studies involving behavioral and mental health, prospective associations with Google and Bing search data series have been aligned with clinical outcomes [18]. In the subsequent sections, we summarize findings from peer-reviewed publications representing research conducted on disease or condition diagnosis using search history to predict future health searches and patient-consented data linked to other health research data. Although these data represent a promising avenue for health research, cohort sizes in studies of internet search data linked with clinical records are typically smaller than those evaluating only individual internet search data, necessitating careful consideration when interpreting results and designing future studies. Nevertheless, studies going beyond aggregate, anonymized data offer important insights, particularly in understanding behavioral and mental health conditions.

### Cancer

#### Overview

The study titled “Patterns of Information-Seeking for Cancer on the Internet: An Analysis of Real World Data” [19] was among the first internet query–based studies to present a detailed analysis of cancer-related internet searches. It analyzed Yahoo search data over 3 months, involving 50,117 users and 225,675 queries [19]. Findings include a correlation between the aggressiveness of the cancer type and the intensity and duration of search patterns [19]. The study used linear regression and hidden Markov models to analyze these patterns [19], finding a stronger focus on treatment information in searches for aggressive cancers, while users demonstrated a higher interest in support groups for less aggressive cancers [19]. This research underscores the potential clinical utility and limitations of using internet search data to understand the information needs of patients with cancer and their acquaintances. It suggests that while such data offer valuable insights, they may not represent the diversity of experiences and needs of patients with cancer [19].

Soldaini and Yom-Tov [20] also demonstrated algorithms designed to identify specific traits of interest in anonymous internet users. These algorithms’ applications in the medical domain demonstrate their effectiveness in identifying potential patients with cancer based on search patterns, predicting disease distributions within a population, and offering valuable insights for early disease screening and epidemiological studies [19-21].

#### Parents of Patients With Pediatric Cancer

A study titled “Health-Related Google Searches Performed by Parents of Pediatric Oncology Patients” [22] analyzed the search behaviors of 98 parents of patients with pediatric cancer and found that parents conducted a higher proportion of health-related searches (13%) compared to the general population (5%) [22]. Searches peaked around key medical events such as diagnosis and treatment phases [22].

Approximately 31% of the searches involved symptoms, disease, and medical information, and 29% involved hospitals and care sites [22]. Cancer-specific searches comprised 18% of the health-related queries [20]. The study emphasized the critical role of the internet in the information-seeking process of parents coping with a child’s cancer diagnosis and treatment and highlighted parents’ reliance on the internet for health care information in pediatric oncology [22]. This reliance underscores the need for accessible, reliable online medical information and indicates potential focus areas for health care providers in patient and family education.

#### Lung Cancer

A study titled “Evaluation of the Feasibility of Screening Patients for Early Signs of Lung Carcinoma in Web Search Logs” [23] explored the use of anonymized search logs for the early detection of lung carcinoma. White and Horvitz [23] used anonymized search logs from Bing involving millions of US. English-speaking users. Of these, 5443 users who later searched for lung carcinoma symptoms were identified as positive cases [23]. Statistical classifiers were used to predict search appearances based on earlier search patterns [23]. Findings showed that certain search behaviors could indicate a higher risk of lung cancer, with true-positive rates ranging from 3% to 57% for different false-positive rates [23]. The study concluded that web search data could aid in early lung cancer detection, highlighting new directions in identifying risk factors and screening opportunities [23].

A study titled “The Role of Web-Based Health Information in Help-Seeking Behavior Prior to a Diagnosis of Lung Cancer: A Mixed-Methods Study” [24] investigated how web-based health information influences diagnosis for patients with lung cancer. Through surveys and interviews, the study captured the experiences and behaviors of patients and their next of kin [24]. Quantitative methods were used to establish the proportion of lung cancer cases in which prediagnosis web searches occurred [24]. Qualitative methods were used to explore individuals’ perceptions of the impact their web searches had on the pathway to diagnosis and the barriers that might prevent individuals from accessing the web for information before the diagnosis [24]. Mixed methods were required, because a survey was needed to screen for relevant individuals for interviews as low levels of web use among patients with lung cancer were expected [24]. Thus, this study included a cross-sectional, retrospective survey and a qualitative interview study with a subsample of the survey participants [24]. It found that 20.4% of participants engaged in prediagnosis web searches, mainly using Google and NHS Direct [24]. These searches played a role in all the 3 intervals leading to diagnosis: symptom appraisal, decision-making for seeking health care, and interaction with health professionals [24]. The study underscores the growing significance of the internet in early disease detection and patient decision-making [24].

#### Ovarian Cancer

A study titled “Using Online Search Activity for Earlier Detection of Gynaecological Malignancy” [25] focuses on leveraging Google search data to predict gynecological cancers, particularly ovarian cancer. Although this study was built upon the research by Soldaini and Yom-Tov [20] that relied on self-identification in queries for outcomes [26], it used clinically verified outcomes to enhance the findings’ robustness and reliability. The study, conducted from December 2020 to June 2022 at a London university hospital, involved 235 women who consented to share their Google search histories [25]. It aimed to distinguish between search patterns of women with malignant diseases and those with benign tumors and to explore the possibility of earlier diagnosis through these patterns [25] and found notable differences in search patterns up to a year before clinical diagnosis, with a predictive model showing an area under the curve (AUC) of 0.82 for individuals who frequently searched for health-related topics [25], demonstrating the potential of using online search data as a supplementary tool for early cancer detection [25]. Chen et al [27] noted that despite the limited datasets in this study, a tendency is apparent toward heightened online search activity before patients with malignant cases visit a general practitioner [27].

#### Pancreatic Cancer

A study titled “Screening for Pancreatic Adenocarcinoma Using Signals From Web Search Logs” [17] explored the use of Bing web search logs to predict pancreatic adenocarcinoma. The study involved 9.2 million US English-speaking users, focusing on the feasibility of the early detection of pancreatic cancer by analyzing search patterns [55]. The researchers analyzed Bing anonymized search logs, looking for patterns that might indicate the early stages of pancreatic adenocarcinoma [17]. They identified users who searched for symptoms or treatment related to pancreatic cancer and then traced their search history backward, looking for early signals of the disease [17]. This retrospective analysis searched for distinctive search patterns before the actual diagnosis [17]. The findings demonstrated the potential of search log analysis to identify early signs of serious illnesses and that certain search behaviors could indicate pancreatic adenocarcinoma, achieving true-positive rates of 5% to 15% with extremely low false-positive rates [17]. This method could complement traditional diagnostic methods and constitutes an innovative approach suggesting a new direction for cancer screening using web search data in health surveillance and early diagnosis [17].

### Cardiovascular Disease

Shaklai et al [28] evaluated the predictive potential of Bing search queries for impending stroke events in an at-risk population in a health care setting in Israel. The study analyzed data from 285 individuals who self-reported a stroke and 1195 controls, focusing on changes in cognitive traits evident in their internet searches [28], and found that certain query attributes related to cognitive function were predictive of an impending stroke [28]. The model showed high accuracy, particularly as the date of the stroke approached, suggesting that monitoring internet search patterns could offer a valuable tool for early stroke detection [28].

### Emergency Department Visits

Asch et al [29] explored the potential of Google search histories in predicting emergency department (ED) visits and their correlation with clinical conditions. The study included 103 participants who consented to share their Google search data collected 7 days before the ED visits; their electronic medical record (EMR) data were included [29]. The analysis of 591,421 unique search queries revealed that 37,469 (6.34%) were health related [29]. In the week before an ED visit, 15% of searches were health related, with many directly related to the participants’ chief concerns [18]. The study highlights the potential of internet search data in anticipating health care use and understanding patients’ health-related concerns [29].

### Mental and Behavioral Health

#### Addiction

Nitzburg et al [30] used internet search data to identify patients seeking drug treatment services for alcohol use disorder. Leveraging internet search data, the study explored how medical symptom queries correlate with subsequent searches about Alcoholics Anonymous and Narcotics Anonymous treatment [30]. Routine visits to primary care physicians serve as initial points of contact for problem drinkers, providing an opportunity to motivate them toward alcohol-reduction treatment. Brief intervention protocols, integrated into routine care, aim to reduce patients’ drinking levels [30]. By analyzing anonymized Bing search data, the study identified common medical symptoms preceding searches for 12-step programs, illuminating potential avenues to enhance brief intervention protocols’ efficacy in motivating individuals toward seeking treatment [30]. Findings suggest that emphasizing long-term medical consequences and immediately discomforting symptoms could enhance motivation for seeking treatment.

#### Anxiety and Depression

Three studies [31-33], focusing on depression and anxiety disorders, present innovative approaches to addressing mental health challenges. Zhang et al [31] explored the potential of using personal online activity histories from search engines such as Google and platforms such as YouTube to detect depressive disorder among US college students. By collecting longitudinal data and using machine learning techniques, the study established correlations between shifts in online behaviors and worsening mental health profiles during the COVID-19 pandemic [31], highlighting the feasibility of leveraging ubiquitous online data for noninvasive surveillance of mental health conditions and offering an alternative to traditional screening methods, especially in times of societal disruption [31].

Zaman et al [32] expanded the investigation to examine the relationship between changes in Google search and YouTube engagement behaviors and the exacerbation of depression and anxiety levels among college students during the COVID-19 pandemic. Through longitudinal data collection and correlation analysis, they identified significant associations (*P*=.03) between deteriorating mental health profiles and shifts in online behavior and provided insights into the potential use of these behavioral changes to predict mental health conditions [32]. These findings underscore the importance of using pervasive online data for real-time monitoring and early intervention in mental health care and offer a cost-effective, scalable approach to complement existing screening methods [32].

In a third study, Zaman et al [33] proposed an alternative method for identifying individuals with anxiety disorders and estimating their anxiety levels using personal online activity histories from YouTube and Google search. By collecting multiple rounds of anonymized data and developing explainable features capturing temporal and contextual aspects of online behaviors, they demonstrated results in detecting anxiety disorders and assessing anxiety levels. This study presents a cost-effective and scalable framework that could be deployed in real-world clinical settings, empower health care providers and therapists with insights into anxiety disorders, and enhance mental health care delivery [33]. These 3 studies highlight how leveraging online data for mental health surveillance and intervention offers new avenues for improving mental health outcomes.

Youngmann et al [34] revealed that individuals exhibit distinct information-seeking behaviors when using searches depending on their anxiety level, which is particularly evident in searches for medical symptoms with potentially life-threatening implications. By analyzing mouse-tracking data and other user interactions, a model was developed to predict user anxiety levels that achieved significant correlation (Kendall’s Tau of 0.48) with the severity of symptoms searched [34]. The findings underscore the importance of incorporating user anxiety information to accurately measure search engine performance, which is crucial in effectively delivering critical medical information and suicide prevention resources.

#### Eating Disorders

Sadeh-Sharvit et al [35] addressed how leveraging internet search data can enable interventions in cases of eating disorders, given their personal and public health costs and the barriers to seeking treatment. By leveraging internet browsing behavior, the study explored whether data from clinically validated online screens can predict the presence of or the high risk for an eating disorder [35]. Results suggest that a machine learning algorithm incorporating variables such as age, search activity related to eating disorders, and internet use patterns can identify women screening positive for eating disorders with moderate accuracy, potentially enabling early intervention to reduce the prevalence of these disorders. The study acknowledges the need for larger sample sizes and inclusion of diverse populations, along with the ethical and privacy concerns in implementing predictive models for eating disorder detection using internet browsing data [35].

#### Intimate Partner Violence

Zaman et al [36] used Google search data to identify intimate partner violence (IPV); 56 participants consented to data analyses that revealed distinctive search characteristics among those with and without IPV experiences [36], suggesting that specific patterns in search behavior, including linguistic attributes and search times, can indicate IPV [36]. These findings highlight the potential use of search data for early detection of and intervention in domestic violence [36].

Youngmann and Yom-Tov [37] analyzed queries from Bing search data involving over 50,000 US individuals experiencing IPV. About half initiated their searches for IPV-related information following an IPV event, while approximately 20% actively concealed their IPV interest [37]. Individuals experiencing IPV showed interest in the effects of IPV, seeking help, ways to escape from abusive situations, and more [37]. This research suggests that while detecting early signs of IPV through search queries may be challenging, even in the later stages of IPV, interventions such as targeted advertisements to assist people in safely leaving violent situations could be highly beneficial [37].

#### Mood Disorders and Suicidality

A study [38] conducted at Northwell Health system included 43 individuals aged between 15 and 30 years with mood disorders who were hospitalized for suicidal thoughts and behaviors and examined their Google search activity before hospitalization. The research identified search patterns related to suicide and behavioral health [38]. A majority (27/43, 63%) conducted suicide-related searches [38]. Participants searched for information that matched their chosen method of attempting suicide in 21% (9/43) of the cases [31]. Suicide-related queries also included unusual suicide methods and references to suicide in popular culture [38]. Most participants (33/43, 77%) used queries related to help-seeking themes, including how to find behavioral health care [38]. Queries related to mood and anxiety symptoms were found among 44% (19/43) of participants and included references to panic disorder, inability to focus, feelings of loneliness, and despair [38]. The results provide insights into digital behaviors of youth with mood disorders facing suicidality, highlighting the potential of internet search data in clinical assessment and intervention strategies [38].

A study titled “Perceived Utility and Characterization of Personal Google Search Histories to Detect Data Patterns Proximal to a Suicide Attempt in Individuals Who Previously Attempted Suicide: Pilot Cohort Study” [39] explored the feasibility and acceptability of using personalized online search data to identify the risk of suicide attempts. It involved 62 participants with a history of suicide attempts and analyzed changes in online search behavior up to 60 days before an attempt, revealing patterns such as increased searches related to suicide methods and expressions of anger [39]. The study highlights the potential of internet search data to identify early warning signs of suicide risk, although participants raised concerns about privacy and accuracy [39].

#### Psychosis

A study titled “Google Search Activity in Early Psychosis: A Qualitative Analysis of Internet Search Query Content in First Episode Psychosis” [18] analyzed Google search queries of individuals before their first hospitalization for psychosis. This qualitative evaluation involved 20 participants who provided access to their Google archive data and identified common themes during emerging illness [18]. Findings revealed that 75% of participants searched for mental health–related information [18]. Most (75%) participants included delusions in their queries [18]. The study concluded that individuals with early psychosis used the internet to understand their symptoms before seeking psychiatric care [18], highlighting the potential for tailoring online resources to improve pathways to care and shorten durations of untreated psychoses [18].

Aref-Adib et al [40] investigated patterns and consequences of online mental health information–seeking behavior among individuals with psychosis and assessed the acceptability of a mobile mental health app. Individuals with psychosis commonly seek online mental health information, which proves beneficial when shared with clinicians [40]. However, when not shared, it can impact health care decisions [40]. The research underscores the need for a collaborative, shared decision-making approach to online health information seeking that includes discussion with clinicians [40]. Findings suggest that individuals with psychosis lead active digital lives, indicating that introducing a mental health app into services may be positively received.

#### Schizophrenia

A study titled “Utilizing Machine Learning on Internet Search Activity to Support the Diagnostic Process and Relapse Detection in Young Individuals With Early Psychosis: Feasibility Study” [41] explored using internet search data to aid in diagnosing relapses in schizophrenia spectrum disorders (SSDs). It involved 42 participants in the Northwell Health System with SSD and 74 healthy volunteers aged between 15 and 35 years [41]. The IRB-approved study analyzed 32,733 time-stamped search queries [41]. Machine learning algorithms were developed to distinguish between individuals with SSD and healthy volunteers and to predict psychotic relapses [41]. Results showed potential for using online search activity as objective data in psychiatric diagnostics and relapse prediction, with classifiers achieving an AUC of 0.74 for diagnosis and an AUC of 0.71 for relapse prediction [41]. Findings include fewer and shorter searches among SSD participants and specific word use patterns related to symptoms [41]. This approach represents a novel method for integrating digital data into mental health monitoring and diagnostics [41].

### Neurodegenerative Diseases

Internet search data have also been used in diagnosing neurodegenerative diseases. Austin et al [42] explored the relationship between internet search behavior and cognitive function in older adults, with a focus on Alzheimer disease. By continuously tracking and analyzing search terms, the authors found that individuals with poorer cognitive function exhibited distinct patterns in their web searches—they used fewer unique terms and less common vocabulary [42]. This suggests that changes in language use during web searches could serve as an early indicator of cognitive decline, thereby potentially enabling treatment before symptoms fully manifest [42].

Youngmann et al [43] developed a machine learning algorithm to screen for Parkinson disease using data from search interactions. By analyzing the textual content of web queries, the classifier identified individuals at high risk for Parkinson disease [43]. Longitudinal follow-up revealed that those identified as positive showed a higher rate of progression in disease-related features [43]. This innovative approach enables large-scale screening for Parkinson and offers insights into disease progression, potentially facilitating early intervention and management.

Yom-Tov et al [44] investigated the potential of internet search interactions in identifying individuals with amyotrophic lateral sclerosis (ALS). By analyzing search query data, the authors developed a model capable of accurately distinguishing individuals with ALS from controls and disease mimics [44]. The prospective validation further supported the approach’s efficacy, indicating its potential as a screening tool to reduce ALS-associated diagnostic delays [44]. These studies highlight the value of harnessing internet search data for early detection of neurodegenerative diseases and offer promising avenues for improving clinical outcomes.

### Nutritional and Metabolic Diseases

The use of internet search data presents a potential avenue for early detection of nutritional and metabolic diseases, such as diabetes. Hochberg et al [45] analyzed Bing search queries from US users to identify symptoms related to diabetes. Through predictive models, the study could distinguish between users diagnosed with diabetes and those querying symptoms associated with diabetes [45]. The models could detect undiagnosed patients with diabetes up to 240 days before they mentioned being diagnosed [45], highlighting the potential of using search data for earlier diagnosis, which is particularly beneficial for conditions such as type 1 diabetes, where early detection is clinically meaningful [45]. In addition, the study suggests the possibility of searches serving as population-wide screening tools and hints at potential further improvement by incorporating additional user-provided data.

Lebwohl and Yom-Tov [46] investigated the use of internet search data to identify symptoms prompting an interest in celiac disease and the gluten-free diet. By analyzing Bing search queries in the United States, the study characterized symptoms and conditions potentially indicating elevated likelihood of subsequent celiac disease diagnosis [46]. The study identified various symptoms queried before celiac-related searches, including diarrhea, headache, anxiety, depression, and attention-deficit/hyperactivity disorder, but the predictive ability of these searches was limited [46]. The study did observe an increase in antecedent searches for symptoms associated with celiac disease, shedding light on its diverse clinical manifestations and the challenges involved in identifying effective case-finding strategies [46]. These findings underscore the complex nature of a celiac disease diagnosis and the potential for leveraging internet search data to enhance understanding and detection of such nutritional disorders.

The following case study involves applications of irritable bowel syndrome in the context of public health information, misunderstanding, and patterns of decision-making by individuals. A study titled “Evidence From Web-Based Dietary Search Patterns to the Role of B12 Deficiency in Non-Specific Chronic Pain: A Large-Scale Observational Study” [47] used a large dataset of internet search patterns to investigate the relationship between vitamin B12 deficiency and chronic pain.

The study explored the role of vitamin B12 in neuropathy and other neuropsychiatric symptoms using internet search patterns as a proxy for dietary habits [47]. Researchers analyzed search data from 8.5 million people in the United States, focusing on searches related to food and B12 deficiency symptoms [47]. Researchers used Bing search data from October 2016 to examine searches for recipes and terms related to chronic pain and B12 deficiency [47], then used a linear classification model to link food consumption data with searches for medical terms, finding a strong correlation between food-related search patterns and actual food consumption [47]. Terms related to neurological disorders were more commonly searched for in conjunction with B12-poor foods [47]. In addition, people who searched for B12-rich foods were less likely to search for medical terms associated with B12 deficiency [47], and the average estimated daily B12 consumption for people who inquired about B12 was 2.407 µg, compared to 2.395 µg for those who did not, indicating a slight but highly correlated relationship [47].

The study suggests that low vitamin B12 intake may be linked to a broader spectrum of neurological disorders than previously thought [47]. It emphasizes the potential of using internet search patterns for large-scale health studies [47]. The researchers recommend further research to explore the clinical significance of these findings and confirm the role of B12 in neuropsychiatric symptoms [47]. They also note the importance of considering different meat sources in assessing dietary B12 intake [47]. This study offers insights into the potential use of internet search data in public health research to understand the relationship between diet and disease symptoms [47].

### Ethical, Legal, and Social Implications

The ethical, legal, and social implications of using internet search data in health research are multifaceted and critical to address. Ethically, the balance between leveraging data for societal benefit and protecting individual privacy is paramount, particularly given the sensitive nature of health-related search queries. Interviewees note challenges in fostering trust between patients and researchers; patient concerns around consent, transparency, and potential misuse of data require robust safeguards and clear communication to ensure that individuals are informed about how their data will be used and about accessible mechanisms to control or recover their shared data, along with demonstrable benefits of data use. Legally, the application of privacy laws such as HIPAA is complicated by the fact that internet search data fall outside traditional health care regulatory frameworks, necessitating new guidelines to address these gaps. Public acceptance hinges on building trust, which involves addressing fears of surveillance, potential discrimination, and misuse of personal data. These considerations underline the importance of interdisciplinary collaboration to develop ethical frameworks and policies that align technological capabilities with societal expectations and legal requirements.

### Cross-Cutting Themes, Lines of Evidence, and Research Gaps

The results of our analysis of the peer-reviewed research for anonymized and nonanonymized research using Bing or Google search data reflect a clearly nascent domain of IT and data research in assisting with diagnosis determinations. Nevertheless, the advances in structured data, large language models (LLMs), search engines, analytic platforms, and expanding research experiences of health service investigators in population health and individual patient research are promising [58]. At present, there is no structured way of designing these types of studies to aid in diagnosis, but among the most visionary applications of search data, one of the applications is to develop disease-specific predictive models for classifying internet search terminologies that may one day be applied in real time for clinical decision-making.

The published research addresses feasibility and clinical efficacy in prospective studies. None of the reviewed studies have addressed clinical utility. However, some studies discussed implications of how analysis tools and predictive models were used, and some described the conceptualization for data representations in clinical health records [31-33]. Should this type of research demonstrate clinical utility, one could envision the development of apps to empower individual patients. Research of rare diseases could benefit from population-level applications, where crowdsourcing of queries could be mined for commonalities and integrated with population data, disease registries, and EMRs. In addition, utilities for identifying patient candidates for clinical trials could be explored.

Researchers publishing results using internet search data are from 2 general health research domains: researchers from large technology companies with proprietary technology that supports internet searches, who have provided methodological innovations that open doors for clinical investigations [19-27,37,38,41], and academic researchers experienced with large dataset analysis for specific health conditions. The research approaches differ in terms of anonymization, integration with EMRs or other data, the size of the groups studied, and applications of methods and tools. Moreover, it seems likely that research that combines important research questions from clinical and academic settings with the technology engineering domains would catalyze promising clinical and public health insights.

Research questions explored using internet search data usually focus on diseases that evolve over time (ie, subacute or chronic diseases) with a wide array of clinical presentations. One challenge across the domains studied using consented, retrospective internet search data involves the risks for bias in methods for consenting, patient donation, and other areas. However, the associations of causal effects through statistical analysis and mathematical examinations in population studies that use anonymous data sources can frame insights that can be evaluated through pilot studies and prospective randomized clinical trials that can address or help minimize the effects of biases.

Several studies integrate datasets from other social media platforms, such as Instagram and X (formerly known as Twitter), while others use Google Takeout or Bing. We found no publications using Google and Microsoft patient data on the same patients, nor any studies using the same analytic algorithms. Future work could examine crossover effects of patient populations using both data sources because the orientation and structure of the datasets differ.

Only 1 study to date has used a prospective data collection approach that enables patients to contribute data from the beginning of their enrollment (KA Comtois, PhD, personal email correspondence, December 26, 2023). It is unclear whether the search patterns differed in patients who donated their data retrospectively versus patients who donated prospectively. The publications we examined provide no details on the mathematical methods used in classifying terms; there appears to be no consensus or best practices for annotating such data. Thus, reproducing study results may be difficult. We found no publications that have made anonymized datasets created from their research available to other researchers for examination. The most detailed descriptive methods publications provide are supplemental data that include search patterns, common terms, and other data classification details. Future research may encourage more open data policies, including the provision of metadata and the descriptive characteristics of the study populations, which would allow others to validate and build on pilot studies that shape hypothetical associations for enabling diagnosis.

While cancer diagnosis was the initial clinical domain of disease diagnosis captured in the early literature [25], the research has broadened to additional areas, including mental and behavioral health [26,27,30-35,38-41]. The ability to obtain search data from patients provides researchers with valuable insights into the patterns of thought, the periodicity of searching patterns, and themes of research. Perhaps the most noteworthy search domain identified involves queries regarding the patient’s intent to harm oneself or others. A series of studies on integrating patients’ online activities and risk-taking behavior are underway to evaluate their utility in understanding patient management applications. In these domains, the clinical utility is less focused on diagnosis than on monitoring the patient’s status for management and on using search data as an integral tool to intervene or make therapeutic changes in clinical regimens. Government or nongovernmental research organizations are sponsoring several of these studies, marking a milestone for nonindustry sponsorship of search data applications [1].

From the articles we reviewed, there appears to be consensus that assistance with infrastructure development would benefit researchers in designing their studies. In this paper, we have summarized the research findings on tools for harnessing massive datasets and enabling their integration with other datasets, including those with EMR data. We also noted a need for broader information about the nature of the available search datasets, best practices for individuals to manage their datasets with researchers, and the conditions under which their data can be shared. Given the concerns regarding data privacy and security for large datasets in the consumer marketplace and the interplay of these data with HIPAA-regulated data in clinical settings, benefit to the researcher and patient advocacy communities could be achieved by establishing resources and best practices to guide future research design, oversight, and patient benefits from the use of their data.

### Researcher Tools

Researchers have created tools to effectively use internet search data, the integration of which has eased the identification of early signs of issues, ensured user privacy, and streamlined the investigative process. Innovation in these tools (Multimedia Appendix 5) allows researchers to be more successful in their searches, as has been the case in other research domains with novel data sources, such as genomic datasets.

The gTAP web application prioritizes data privacy and security by allowing participants to download their data without sharing personal account credentials, ensuring a higher level of user trust and confidentiality [39]. This feature encourages participation in studies involving symptom analysis and diagnostics, fostering collaboration between researchers and users while maintaining data integrity [39].

Linguistic Inquiry and Word Count, a text analysis software package, exhibits remarkable potential in differentiating linguistic attributes within search logs [36]. By identifying linguistic patterns indicating emotional, sexual, or physical abuse, Linguistic Inquiry and Word Count is instrumental in early symptom identification, providing valuable insights for health care professionals and researchers [36].

The Google natural language processing application programming interface is pivotal in ensuring data privacy and anonymization [48]. By automatically detecting and removing personally identifying information from search history data before they are saved as research data, this application programming interface safeguards the confidentiality of individual study participants [24], enabling researchers to delve into symptom analysis and diagnostics using real-world data while upholding ethical standards and privacy regulations [49].

CrowdTangle, a powerful tool from Meta, aids in monitoring, analyzing, and reporting social media activities [7]. It effectively offers transparency across various social media platforms, positioning it as an invaluable resource for understanding public discourse and sentiment regarding health-related symptoms and conditions [7]. It is the most effective transparency tool in the history of social media [15].

Latent Dirichlet allocation and Differential Language Analysis Toolkit are cutting-edge methodologies in text analysis [59]. Latent Dirichlet allocation produces clusters of words that occur in the same context across Facebook posts, yielding semantically coherent topics [15]. Differential Language Analysis Toolkit determines the relative frequency with which users use words (ie, unigrams) and 2-word phrases (ie, bigrams) and can also retain variables and phrases [59]. Both are pivotal in the identification of potential symptoms or health-related discussions [59].

In a recent diagnostic study evaluating artificial intelligence (AI) capabilities, the use of the AI chatbot GPT-4 (OpenAI) showcased remarkable proficiency in certain diagnostic scenarios [60]. Comparing the LLM’s performance with a broad survey of human clinicians, the study revealed that the LLM surpassed human clinicians in accurately determining pretest and posttest probabilities following a negative test result across 5 cases, although the performance was less robust after positive test results [60]. The study suggests that leveraging probabilistic recommendations from such LLMs could enhance human diagnostic capabilities and that combining AI’s probabilistic, narrative, and heuristic diagnostic approaches could improve diagnostic accuracy [60].

These tools offer greater accuracy and prioritize user privacy and data security. Integrating them into research and health care systems enables early detection and better understanding of symptoms and contributes to well-being outcomes, especially for older individuals, when combined with a comprehensive support system. As technology evolves, these tools are poised to play an increasingly vital role in enhancing health care and advancing diagnostic capabilities.

### Challenges

The use of internet search data to facilitate medical diagnosis faces challenges, including bias, data privacy, and misinformation. The ethical use of patient data is crucial. The study by Wachter and Mittelstadt [61] titled “A Right to Reasonable Interferences: Rethinking Data Protection Law in the Age of Big Data and AI” analyzes ethical dilemmas surrounding the use of big data in health care, emphasizing the need to balance patient privacy with the benefits of big data analytics [41] and the importance of consent and transparency in the collection and use of patient data. It also highlighted biases and inequalities resulting from mismanaged data practices [61]. Yom-Tov and Cherlow [50] further emphasized the need to carefully consider the ethical implications and suggest solutions that balance the benefits and challenges of online screening services.

In our exploration of the field of information sciences concerning internet search data, a notable challenge emerged—the lack of infrastructure for constructing a robust analytic approach to leverage these data in medical and health services research. Thus, we investigated alternative open data research organizational models and discovered the pioneering work of Lane [62]. In her book *Democratizing Our Data: A Manifesto* [62], Lane introduces an organizational model to revolutionize data accessibility and usefulness. Within this context, the Institute for Research on Innovation and Science (IRIS) stands out with its groundbreaking contribution, the Universities: Measuring the Impacts of Research on Innovation, Competitiveness, and Science (UMETRICS) dataset [62]. UMETRICS constitutes a burgeoning research asset, harnessing administrative data—information collected primarily for administrative purposes, such as record keeping, then repurposed for research to analyze health care patterns—from 30 prominent universities that collectively contribute over one-third of federal research and development spending in academia [62]. This dataset signifies a shift in data practices, fundamentally reshaping data collection methodologies, fortifying privacy safeguards, and fostering the generation of new products [62]. Along with pioneering the inception of “big data” social science research infrastructures, IRIS confronted the formidable challenge of comprehending the impact of research funding on scientific and economic activities, doing so by spearheading construction of an entire infrastructure for tracing the effects of research funding on individuals and interconnected networks [62]. IRIS developed a highly adaptable data infrastructure, composed of a decentralized network of federal agencies responsible for collecting, analyzing, and disseminating data on various aspects of the country, which caters directly to research universities and provides impactful methods to assess scientific and economic implications of their research pursuits, surpassing the federal statistical system [62]. Critical to IRIS’s approach was establishing a data infrastructure rooted in transparent governance, robust privacy protocols, and effective confidentiality protections, buttressed by a sustainable business model requiring contributions from data providers and sponsored projects [62]. This comprehensive approach provides an important foundation and framework for transformative data activities in the realm of social media and promises accessible and purposeful data use [62].

Leveraging the work of such organizations may unify researchers’ approaches in governance, transparency, data sharing, and related aspects essential for using search data effectively. Integrating these insights into our analysis could illuminate pathways to address critical gaps in this field. We need to establish robust infrastructures that equip researchers with the necessary tools and resources to delve into such research at scale. Assessing the true utility of search data in medical diagnosis requires comprehensive frameworks that facilitate large-scale analysis while ensuring data privacy and integrity. Moreover, should research demonstrate the valuable application of these findings, such infrastructures will play a pivotal role in translating discoveries into actionable insights for clinical practice and health care policy.

### Health Care Practice and Policy Implications

The integration of internet search data with health research datasets suggests profound implications for health care practice and policy, necessitating careful ethical and technical considerations. Such research poses unique challenges, beyond the scope of traditional regulatory frameworks such as HIPAA. While HIPAA governs the use and disclosure of protected health information held by covered entities, it may not fully address the intricacies of internet search data, which contain a wealth of information about individuals’ health behaviors and concerns and potentially sensitive details not captured by conventional health records.

In the context of policy implications, IRBs play a crucial role in ensuring ethical research practices and safeguarding participants’ welfare. For research involving internet search data, IRBs must navigate the nuanced landscape of privacy, consent, and potential risks. Since internet search data may not fall under the strict purview of HIPAA, it is essential that IRBs establish clear guidelines for these data.

### Research Directions

The findings of this literature review underscore the need for concerted efforts to stimulate research and fully explore the potential clinical utility of integrating internet search data with health research datasets. While our review did not identify a clear clinical utility, it revealed promising dimensions in behavioral health, early rare disease detection, and cancer diagnoses. The limited research in this domain since the seminal work of White and Horvitz [51,52] in 2013 and 2014 and its relative scarcity suggest potential barriers related to researchers’ familiarity with the data technical complexities in mining the data, or other yet-to-be-identified obstacles [53,54].

To address these gaps and challenges, we propose a multifaceted approach in 4 key areas. First, there is an urgent need to assess the value and utility of internet search and activity datasets in conjunction with health research datasets, including clinical records. This evaluation should explore how such integration can enhance the diagnostic process, contribute to early disease detection, provide personalized health insights, inform data-driven decision-making, and improve patient experiences.

Second, future research should focus on mental health, autism, attention-deficit/hyperactivity disorder, and chronic or rare diseases. Tailoring projects to address unique diagnostic and treatment challenges may involve creating customized algorithms and tools that cater to these patients’ needs and nuanced health conditions.

Third, the introduction of innovative analytics, including advanced machine learning and AI models, should be a priority [63]. These sophisticated techniques can uncover hidden patterns and tendencies within the integrated datasets, offering a new frontier in diagnostic accuracy. Developing predictive models could revolutionize health care delivery by providing more precise insights into patient conditions and optimizing treatment plans [64,65]. The advancement of infrastructure platforms that could aggregate search data with other types of online data (ie, social media and generative AI) and clinical data would allow for this research to be conducted at scale and for the introduction of the kind of innovative analytics described earlier [65,66].

Fourth, enhancing patient involvement in modernizing the consent process is paramount. Research should focus on developing innovative strategies that streamline and modernize consent, prioritizing transparency, trust, and patient comfort with the use of their data. Involving patients in shaping research practices ensures ethical, patient-centered health care research, reduces administrative burdens, and promotes accessibility and efficiency.

This comprehensive effort aims to propel research in this field, overcoming current limitations and paving the way for transformative applications of internet search and activity data in health care diagnostics. Innovations, such as reusable platforms for consent and data collection, may improve researchers and patient engagement. Standardized platforms that streamline the consent process and facilitate data collection can substantially enhance efficiency and scalability. These platforms should incorporate user-friendly interfaces, clear consent language, and robust security measures to ensure privacy compliance and promote patient trust. Reusable frameworks expedite research, reduce administrative burdens, and foster collaboration, advancing our understanding of the clinical utility of internet search data in medical diagnosis.

Respecting patient privacy and obtaining informed consent are foundational principles in health care research. Because the integration of internet search data involves potentially sensitive information, careful attention must be paid to ethical considerations. Transparent and user-friendly consent models are needed to ensure that patients understand who will have access to their data and how their data will be used. Innovative approaches to patient engagement should prioritize educating individuals about the benefits and risks of contributing their internet search data to research. In addition, robust security measures and compliance with privacy regulations are imperative to protect patient confidentiality. Policy makers are pivotal in establishing clear guidelines and regulations that balance the potential benefits of research using internet search data and patients’ medical data with the imperative to uphold patient rights and privacy. Striking the right balance between facilitating research advancements and safeguarding patient interests is critical for the responsible and ethical use of internet search data in health care practice and policy.

### Conclusions

In today’s health care system, many patients lack access to timely, accurate diagnoses, missing the benefits of early detection and treatment, leading to suboptimal outcomes, disparities, and economic impacts. Advances in engineering, computing, social science, and data analytics are yielding transformative insights for public health and clinical medicine. Recently, the convergence of these advancements, particularly in LLMs and generative AI, has captured the imagination of health professionals and the public.

Initial studies have highlighted approaches involving study design, technical innovation, and data management to explore the potential of leveraging internet search data alongside clinical data for early diagnosis. Further research is needed to validate assumptions from search history studies and assess their utility in case-control studies or small cohorts linking symptoms to outcomes. Implementation studies are also necessary to establish the clinical utility of these strategies in real-world settings. Challenges include the costs of diagnostic assessments, particularly for low-frequency conditions or those with high false-positive rates and medical risks. No framework currently exists for integrating internet search queries into clinical assessments, raising questions such as how to distinguish chronic from rare disease indicators.

Globally, people extensively use internet searches for symptom understanding, self-diagnosis, and treatment, creating a vast volume of health-related queries. An individual’s search history is a valuable data source offering insights into their diagnostic journey, enabling researchers to track symptoms and predict conditions. Linking search data with health care use information reveals disparities by factors such as insurance, race, and education. Empowering patients to understand and leverage these data can enhance diagnosis. However, critical issues—epidemiological, privacy, consent, infrastructure, and validation—remain pivotal for advancing this research.

The Gordon and Betty Moore Foundation’s Diagnostic Excellence Initiative is a step toward a future in which health care is more accessible, patient centric, and driven by IT and data. The field continues to evolve, promising a healthier, more informed society.

The interrogation of search data is in its infancy. Initial studies have identified the promise of using search data for personal health and population health benefits, including aiding medical diagnoses. While the clinical utility of enabling health care professionals to apply powerful analytic engines to specific diagnoses has yet to be attained, research into achieving this goal is accelerating rapidly. This analysis points to the need for strategic and tactical measures by health services researchers, technology engineers, policy makers, and regulators to advance this research, optimize its social good, and prevent harm or misuse.

## Appendix

Multimedia Appendix 1 Search term results.

Multimedia Appendix 2 PRISMA (Preferred Reporting Items for Systematic Reviews and Meta-Analyses) checklist.

Multimedia Appendix 3 Survey instrument for informational interviews on internet search data in health research.

Multimedia Appendix 4 Literature matrix.

Multimedia Appendix 5 Tools developed to assist researchers in the use of search data.

## Abbreviations

AI: artificial intelligence
ALS: amyotrophic lateral sclerosis
AUC: area under the curve
ED: emergency department
EMR: electronic medical record
HIPAA: Health Insurance Portability and Accountability Act
IPV: intimate partner violence
IRB: institutional review board
IRIS: Institute for Research on Innovation and Science
LLM: large language model
PRISMA: Preferred Reporting Items for Systematic Reviews and Meta-Analyses
SSD: schizophrenia spectrum disorder
UMETRICS: Universities: Measuring the Impacts of Research on Innovation, Competitiveness, and Science

## Data Availability

All data generated or analyzed during this study are included in this published article (and its supplementary information files).

## References

[1] Research on pre-hospital diagnostic delay. AcademyHealth.

[2] Eysenbach G (2000). A framework for evaluating e-health: systematic review of studies assessing the quality of health information and services for patients on the Internet. J Med Internet Res.

[3] Bianchi T Global market share of leading desktop search engines 2015-2023. Statista.

[4] Farr C Your Google searches can be used to predict when you're about to go to the emergency room, researchers find. CNBC.

[5] Healthcare marketing SEO during the pandemic and beyond. Milestone.

[6] Hochberg I, Allon R, Yom-Tov E (2020). Assessment of the frequency of online searches for symptoms before diagnosis: analysis of archival data. J Med Internet Res.

[7] Ahmed A Google is still not the all-knowing, almighty search engine as 15 percent of queries are ‘never seen before’ by tech giant. Digital Information World.

[8] Tang H, Ng JH (2006). Googling for a diagnosis--use of Google as a diagnostic aid: internet based study. BMJ.

[9] Ayers JW, Chu B, Zhu Z, Leas EC, Smith DM, Dredze M, Broniatowski DA (2021). Spread of misinformation about face masks and COVID-19 by automated software on Facebook. JAMA Intern Med.

[10] Chang S, Fourney A, Horvitz E Accurate measures of vaccination and concerns of vaccine holdouts from web search logs. arXiv.

[11] Diaz P, Reddy P, Ramasahayam R, Kuchakulla M, Ramasamy R (2021). COVID-19 vaccine hesitancy linked to increased internet search queries for side effects on fertility potential in the initial rollout phase following Emergency Use Authorization. Andrologia.

[12] Larson HJ, Broniatowski DA (2021). Volatility of vaccine confidence. Science.

[13] Abroms LC, Koban D, Krishnan N, Napolitano M, Simmens S, Caskey B, Wu T, Broniatowski DA (2024). Empathic engagement with the COVID-19 vaccine hesitant in private Facebook groups: a randomized trial. Health Educ Behav.

[14] Gu J, Dor A, Li K, Broniatowski DA, Hatheway M, Fritz L, Abroms LC (2022). The impact of Facebook's vaccine misinformation policy on user endorsements of vaccine content: an interrupted time series analysis. Vaccine.

[15] Broniatowski DA, Dredze M, Ayers JW (2021). "First do no harm": effective communication about COVID-19 vaccines. Am J Public Health.

[16] Gianfredi V, Provenzano S, Santangelo OE (2021). What can internet users' behaviours reveal about the mental health impacts of the COVID-19 pandemic? A systematic review. Public Health.

[17] Paparrizos J, White RW, Horvitz E (2016). Screening for pancreatic adenocarcinoma using signals from web search logs: feasibility study and results. J Oncol Pract.

[18] Kirschenbaum MA, Birnbaum ML, Rizvi A, Muscat W, Patel L, Kane JM (2020). Google search activity in early psychosis: a qualitative analysis of internet search query content in first episode psychosis. Early Interv Psychiatry.

[19] Ofran Y, Paltiel O, Pelleg D, Rowe JM, Yom-Tov E (2012). Patterns of information-seeking for cancer on the internet: an analysis of real world data. PLoS One.

[20] Soldaini L, Yom-Tov E Inferring individual attributes from search engine queries and auxiliary information. arXiv.

[21] Yom-Tov E (2020). Screening for cancer using a learning internet advertising system. ACM Trans Comput Healthcare.

[22] Phillips CA, Hunt A, Salvesen-Quinn M, Guerra J, Schapira MM, Bailey LC, Merchant RM (2019). Health-related Google searches performed by parents of pediatric oncology patients. Pediatr Blood Cancer.

[23] White RW, Horvitz E (2017). Evaluation of the feasibility of screening patients for early signs of lung carcinoma in web search logs. JAMA Oncol.

[24] Mueller J, Jay C, Harper S, Todd C (2017). The role of web-based health information in help-seeking behavior prior to a diagnosis of lung cancer: a mixed-methods study. J Med Internet Res.

[25] Barcroft JF, Yom-Tov E, Lampos V, Ellis LB, Guzman D, Ponce-López V, Bourne T, Cox IJ, Saso S (2024). Using online search activity for earlier detection of gynaecological malignancy. BMC Public Health.

[26] Soldaini L, Yates A, Yom-Tov E, Frieder O, Goharian N (2015). Enhancing web search in the medical domain via query clarification. Inf Retrieval J.

[27] Chen G, Xie J, Zhang Y, Yang M, Xie Y, Hou W, Zhang Z, Zhang X, Zhang J, Chen Y, Liao W, Liu B, Zhang JJ, Wang Y (2022). Identification of pathological types of adnexal masses from ultrasound images using deep learning models. Ultrasound Obstet Gynecol.

[28] Shaklai S, Gilad-Bachrach R, Yom-Tov E, Stern N (2021). Detecting impending stroke from cognitive traits evident in internet searches: analysis of archival data. J Med Internet Res.

[29] Asch JM, Asch DA, Klinger EV, Marks J, Sadek N, Merchant RM (2019). Google search histories of patients presenting to an emergency department: an observational study. BMJ Open.

[30] Nitzburg G, Weber I, Yom-Tov E (2019). Internet searches for medical symptoms before seeking information on 12-step addiction treatment programs: a web-search log analysis. J Med Internet Res.

[31] Zhang B, Zaman A, Acharyya R, Hoque E, Silenzio V, Kautz H Detecting individuals with depressive disorder from personal Google search and YouTube history logs. arXiv.

[32] Zaman A, Zhang B, Silenzio V, Kautz H, Hoque E (2020). The relationships of deteriorating depression and anxiety with longitudinal behavioral changes in Google and YouTube use during COVID-19: observational study. JMIR Ment Health.

[33] Zaman A, Zhang B, Silenzio V, Hoque E, Kautz H Individual-level anxiety detection and prediction from longitudinal YouTube and Google search engagement logs. arXiv.

[34] Youngmann B, Yom-Tov E (2018). Anxiety and information seeking: evidence from large-scale mouse tracking. Proceedings of the 2018 World Wide Web Conference.

[35] Sadeh-Sharvit S, Fitzsimmons-Craft EE, Taylor CB, Yom-Tov E (2020). Predicting eating disorders from internet activity. Int J Eat Disord.

[36] Zaman A, Kautz H, Silenzio V, Hoque ME, Nichols-Hadeed C, Cerulli C (2021). Discovering intimate partner violence from web search history. Smart Health.

[37] Youngmann B, Yom-Tov E (2022). Intimate partner violence as reflected in internet search data. Soc Sci Comput Rev.

[38] Moon KC, Van Meter AR, Kirschenbaum MA, Ali A, Kane JM, Birnbaum ML (2021). Internet search activity of young people with mood disorders who are hospitalized for suicidal thoughts and behaviors: qualitative study of google search activity. JMIR Ment Health.

[39] Areán PA, Pratap A, Hsin H, Huppert TK, Hendricks KE, Heagerty PJ, Cohen T, Bagge C, Comtois KA (2021). Perceived utility and characterization of personal Google search histories to detect data patterns proximal to a suicide attempt in individuals who previously attempted suicide: pilot cohort study. J Med Internet Res.

[40] Aref-Adib G, O'Hanlon P, Fullarton K, Morant N, Sommerlad A, Johnson S, Osborn D (2016). A qualitative study of online mental health information seeking behaviour by those with psychosis. BMC Psychiatry.

[41] Birnbaum ML, Kulkarni P, Van Meter A, Chen V, Rizvi AF, Arenare E, De Choudhury M, Kane JM (2020). Utilizing machine learning on internet search activity to support the diagnostic process and relapse detection in young individuals with early psychosis: feasibility study. JMIR Ment Health.

[42] Austin J, Hollingshead K, Kaye J (2017). Internet searches and their relationship to cognitive function in older adults: cross-sectional analysis. J Med Internet Res.

[43] Youngmann B, Allerhand L, Paltiel O, Yom-Tov E, Arkadir D (2019). A machine learning algorithm successfully screens for Parkinson's in web users. Ann Clin Transl Neurol.

[44] Yom-Tov E, Navar I, Fraenkel E, Berry JD (2024). Identifying amyotrophic lateral sclerosis through interactions with an internet search engine. Muscle Nerve.

[45] Hochberg I, Daoud D, Shehadeh N, Yom-Tov E (2019). Can internet search engine queries be used to diagnose diabetes? Analysis of archival search data. Acta Diabetol.

[46] Lebwohl B, Yom-Tov E (2019). Symptoms prompting interest in celiac disease and the gluten-free diet: analysis of internet search term data. J Med Internet Res.

[47] Giat E, Yom-Tov E (2018). Evidence from web-based dietary search patterns to the role of b12 deficiency in non-specific chronic pain: a large-scale observational study. J Med Internet Res.

[48] Zaman A, Acharyya R, Kautz H, Silenzio V (2019). Detecting low self-esteem in youths from web search data. Proceedings of the 2019 Conference on World Wide Web.

[49] Zaman, Anis Combining traditional and non-traditional data stream for understanding mental health. University of Rochester Libraries.

[50] Yom-Tov E, Cherlow Y (2020). Ethical challenges and opportunities associated with the ability to perform medical screening from interactions with search engines: viewpoint. J Med Internet Res.

[51] White RW, Horvitz E (2014). From health search to healthcare: explorations of intention and utilization via query logs and user surveys. J Am Med Inform Assoc.

[52] White R, Horvitz E (2013). From web search to healthcare utilization: privacy-sensitive studies from mobile data. J Am Med Inform Assoc.

[53] Yom-Tov E, White RW, Horvitz E (2014). Seeking insights about cycling mood disorders via anonymized search logs. J Med Internet Res.

[54] Cohen Zion M, Gescheit I, Levy N, Yom-Tov E (2022). Identifying sleep disorders from search engine activity: combining user-generated data with a clinically validated questionnaire. J Med Internet Res.

[55] Phillips CA, Barz Leahy A, Li Y, Schapira MM, Bailey LC, Merchant RM (2018). Relationship between state-level Google online search volume and cancer incidence in the United States: retrospective study. J Med Internet Res.

[56] Ginsberg J, Mohebbi MH, Patel RS, Brammer L, Smolinski MS, Brilliant L (2009). Detecting influenza epidemics using search engine query data. Nature.

[57] 2018 requirements (2018 common rule). Office for Human Research Protections, U.S. Department of Health and Human Services.

[58] McDuff D, Schaekermann M, Tu T, Palepu A, Wang A, Garrison J, Singhal K, Sharma Y, Azizi S Towards accurate differential diagnosis with large language models. arXiv.

[59] Eichstaedt JC, Smith RJ, Merchant RM, Ungar LH, Crutchley P, Preoţiuc-Pietro D, Asch DA, Schwartz HA (2018). Facebook language predicts depression in medical records. Proc Natl Acad Sci U S A.

[60] Rodman A, Buckley TA, Manrai AK, Morgan DJ (2023). Artificial intelligence vs clinician performance in estimating probabilities of diagnoses before and after testing. JAMA Netw Open.

[61] Wachter S, Mittelstadt B (2019). A right to reasonable interferences: re-thinking data protection law in the age of Big Data and AI. Colum Bus L Rev. Colum Bus L Rev.

[62] Lane J (2021). Democratizing Our Data: A Manifesto.

[63] Beam AL, Drazen JM, Kohane IS, Leong TY, Manrai AK, Rubin EJ (2023). Artificial intelligence in medicine. N Engl J Med.

[64] Majerowicz A, Tracy S (2010). Telemedicine. Bridging gaps in healthcare delivery. J AHIMA.

[65] Greenes RA, Bushko RG (2009). Informatics and a health care strategy for the future - general directions. Studies in Health Technology and Informatics.

[66] Strengers Y, Duque M, Mortimer M, Pink S, Nicholls L, Horan B, Eugene A, Thomson S (2022). “Isn't this marvelous”: supporting older adults’ wellbeing with smart home devices through curiosity, play and experimentation. Proceedings of the 2022 ACM Designing Interactive Systems Conference.

