# Supplementary material for: Harnessing Internet Search Data as a Potential Tool for Medical Diagnosis: Literature Review
Source: JMIR Ment Health. 2025 Feb 11;12:e63149. doi: 10.2196/63149 (PMC11862766; doi:10.2196/63149)
Supplement: Multimedia Appendix 1 [file mental_v12i1e63149_app1.docx]

Appendix 1: Search Term Results

| **Term** | **PubMed Results** |
| --- | --- |
| "internet search" AND diagnosis | 443 |
| "online search" AND diagnosis | 41 |
| "search engine" AND diagnosis | 124 |
| "web search" AND diagnosis | 13 |
| "search behavior" AND diagnosis | 9 |
| (Google OR Bing) AND diagnosis | 1,566 |
| Takeout AND diagnosis | 4 |
| "internet" AND "early diagnosis" | 40 |
| "internet search" AND disease detection | 39 |
| "online search" AND disease detection | 4 |
| "search engine" AND disease detection | 18 |
| "web search" AND disease detection | 20 |
| "search behavior" AND disease detection | 7 |
| (Google OR Bing) AND disease detection | 580 |
| Takeout AND disease detection | 3 |
| internet AND "disease detection" | 111 |
|  |  |
| "internet search" AND disease identification | 19 |
| "online search" AND disease identification | 31 |
| "search engine" AND disease identification | 124 |
| "web search" AND disease identification | 10 |
| "search behavior" AND disease identification | 1 |
| (Google OR Bing) AND disease identification | 1,194 |
| Takeout AND disease identification | 3 |
|  |  |
| "internet search" AND diagnostic accuracy | 34 |
| "online search" AND diagnostic accuracy | 23 |
| "search engine" AND diagnostic accuracy | 140 |
| "web search" AND diagnostic accuracy | 15 |
| "search behavior" AND diagnostic accuracy | 12 |
| (Google OR Bing) AND diagnostic accuracy | 1,799 |
| Takeout AND diagnostic accuracy | 0 |
