# Supplementary material for: Harnessing Internet Search Data as a Potential Tool for Medical Diagnosis: Literature Review
Source: JMIR Ment Health. 2025 Feb 11;12:e63149. doi: 10.2196/63149 (PMC11862766; doi:10.2196/63149)
Supplement: Multimedia Appendix 3 [file mental_v12i1e63149_app3.docx]

Appendix 3: Survey Instrument for Informational Interviews on Internet Search Data in Health Research

# Introduction:

Thank you for participating in this study on the use of internet search data in health research. Your insights will help us better understand the opportunities and challenges in this field. This interview will focus on the user of internet search data in applications in health research, ethical considerations, privacy and security issues, and public acceptance. Your responses will remain confidential and will be used only for research purposes.

# Section 1: Background Information

1. **Professional Role**
   - Researcher
   - Ethicist
   - Patient Advocate
   - Open Data Expert
   - Other (please specify):

# Field of Expertise

- - Health Research
  - Data Privacy/Security
  - Open Data Policy
  - Public Health
  - Other (please specify):

# Years of Experience in Your Field

- - Less than 5 years
  - 5–10 years
  - 10–15 years
  - More than 15 years

# Section 2: Applications of Internet Search Data in Health Research

1. What do you perceive as the most promising applications of internet search data in health research?

(Open-ended)

1. Which diseases or conditions do you believe are most suited for early diagnosis or monitoring through internet search data?

(Select all that apply)

- - Cancer
  - Mental and Behavioral Health
  - Neurodegenerative Disorders
  - Nutritional and Metabolic Diseases
  - Other (please specify):

1. In your experience, what are the key challenges in integrating internet search data with health research datasets?

(Open-ended)

# Section 3: Privacy, Security, and Ethical Considerations

1. What are the primary privacy concerns related to using internet search data for health research?

(Open-ended)

1. How do you think researchers can balance the need for data privacy with the potential benefits of using search data in diagnostics?

(Open-ended)

1. How would you rate the current public understanding of privacy risks associated with internet search data in health research?
   - Poor
   - Fair
   - Good
   - Excellent
2. What safeguards or policies would you recommend to protect patient privacy while leveraging internet search data?

(Open-ended)

# Section 4: Public Acceptance and Perception

1. How do you think the public perceives the use of their internet search data in health research?

(Open-ended)

1. What factors do you believe influence public acceptance of this type of research? (Select all that apply)
   - Transparency about data use
   - Clear consent processes
   - Demonstrated health benefits
   - Strong data security measures
   - Other (please specify):
2. What outreach or education strategies would help improve public acceptance? (Open-ended)

# Section 5: Future Directions and Recommendations

1. What are the most critical areas for future research in using internet search data in health research?

(Open-ended)

1. What infrastructure or resources would you recommend to advance this field? (Open-ended)
2. What role do you think interdisciplinary collaboration (e.g., among researchers, ethicists, and patient advocates) should play in shaping this field?

(Open-ended)

# Closing:

Thank you for sharing your insights. If you have any additional comments or recommendations, please share them below:

(Open-ended)
