# Supplementary material for: Harnessing Internet Search Data as a Potential Tool for Medical Diagnosis: Literature Review
Source: JMIR Ment Health. 2025 Feb 11;12:e63149. doi: 10.2196/63149 (PMC11862766; doi:10.2196/63149)
Supplement: Multimedia Appendix 4 [file mental_v12i1e63149_app4.docx]

**Appendix 4: Literature Matrix**

| **Reference Details** | **Brief Synopsis** |
| --- | --- |
| Areán PA, Pratap A, Hsin H, Huppert TK, Hendricks KE, Heagerty PJ, Cohen T, Bagge C, Comtois KA. Perceived utility and characterization of personal Google search histories to detect data patterns proximal to a suicide attempt in individuals who previously attempted suicide: pilot cohort study. J Med Internet Res 2021 May 06; 23(5):e27918. PMID: 33955838 | The aim in conducting this research was to assess the viability and approval of utilizing individualized online information-seeking actions to identify the likelihood of suicide attempts. Variations in online search habits could serve as a viable and permissible method for detecting the risk of suicide. A personalized examination of online information-seeking conduct revealed significant alterations in search behaviors and search phrases associated with early indicators of suicide, noticeable within the period spanning from 2 months to 7 days prior to a suicide attempt. |
| Aref-Adib G, O'Hanlon P, Fullarton K, Morant N, Sommerlad A, Johnson S, Osborn D. A qualitative study of online mental health information seeking behaviour by those with psychosis. BMC Psychiatry 2016 Jul 11; 16:232. PMID: 27400874 | This study delved into the patterns and consequences of online mental health information-seeking behavior among individuals with psychosis and assesses the acceptability of a mobile mental health application (app). Individuals with psychosis commonly seek mental health information online, which proves beneficial when shared collaboratively with clinicians. However, when not shared, it can impact healthcare decisions. The research underscores the necessity for a collaborative approach to online health information seeking, with mental health clinicians encouraging patients to discuss online findings as part of shared decision-making. Findings suggest that individuals with psychosis lead active digital lives, indicating  potential positive reception for the introduction of a mental health app into services. |
| Asch JM, Asch DA, Klinger EV, Marks J, Sadek N, Merchant RM. Google search histories of patients presenting to an emergency department: an observational study. BMJ Open 2019 Feb 20; 9(2):e024791. PMID: 30787088 | The aim of the study was to assess patients’ readiness to share and connect their previous Google search records with information from their electronic medical records (EMRs, and to investigate correlations between search histories and clinical conditions. Out of all searches made within 7 days before an emergency department (ED) visit, 5% were related to health. Among the participants who utilized Google in the week leading up to their visit to the ED, 53% sought information directly linked to their primary health concern. The fluctuations in both the quantity and substance of search activity before an ED visit indicate that there are possibilities to predict and enhance healthcare utilization before such visits occur. Also, patients showed a willingness to grant researchers simultaneous access to their Google search histories and EMR data. |
| Austin J, Hollingshead K, Kaye J. Internet searches and their relationship to cognitive function in older adults: cross-sectional analysis. J Med Internet Res 2017 Sept 06; 19(9):e307. PMID: 28877864 | Internet searches were correlated with cognitive decline. The results suggest that early decline in cognitive function may be detected from the terms people search for when they use the Internet. By continuously tracking the basic aspects of Internet search terms, it may be possible to detect cognitive decline earlier than currently possible, thereby enabling proactive treatment and intervention. |
| Barcroft JF, Yom-Tov E, Lampos V, Ellis LB, Guzman D, Ponce-López V, Bourne T, Cox IJ, Saso S. Using online search activity for earlier detection of gynaecological malignancy. BMC Public Health 2024 Mar 11; 24(1):608. PMID: 38462622 | This study examined the potential of online search data to detect gynaecological cancer in individuals with confirmed diagnoses, suggesting that differences in search patterns were noticeable as early as 360 days before primary care referral. Using a classification model, the study achieved its highest accuracy in predicting cancer risk using data from 60 days before referral, particularly in individuals who frequently searched for health-related topics online. The findings suggest that online search data could offer personalized risk profiles for gynaecological cancer, offering a complementary approach to conventional screening methods and potentially aiding in the earlier detection of various conditions, including cancer. |
| Birnbaum ML, Kulkarni P, Van Meter A, Chen V, Rizvi AF, Arenare E, De Choudhury M, Kane JM. Utilizing machine learning on internet search activity to support the diagnostic process and relapse detection in young individuals with early psychosis: feasibility study. JMIR Ment Health 2020 Sept 01; 7(9):e19348. PMID: 32870161 | The primary objective of this study was to create computational algorithms utilizing internet search patterns, aiming to aid diagnostic processes and recognize potential relapses among individuals diagnosed with schizophrenia spectrum disorders. The research revealed discernible distinctions in the timing, frequency, and nature of online search behavior among young individuals (ages 15-35) with schizophrenia spectrum disorders when compared to their healthy counterparts. Furthermore, alterations in language use and behavioral patterns were detected in the month preceding a relapse leading to hospitalization in individuals diagnosed with schizophrenia spectrum disorders. The study suggests that online search activity holds potential as a means to gather objective and easily accessible markers of psychiatric symptoms. The integration of search behavior as supplementary information related to behavioral health could represent a significant advancement in utilizing unbiased digital data to enhance the monitoring of mental health. |
| Chen G, Xie J, Zhang Y, Yang M, Xie Y, Hou W, Zhang Z, Zhang X, Zhang J, Chen Y, Liao W, Liu B, Zhang JJ, Wang Y. Identification of pathological types of adnexal masses from ultrasound images using deep learning models. Ultrasound Obstet Gynecol 2022; 60(S1):32. DOI: 10.1002/uog.25071 | This research assessed deep-learning models to distinguish between benign, malignant, and borderline adnexal masses. Additionally, the study aimed to classify 15 pathological types based on ultrasound images. This ongoing preliminary investigation has indicated the acceptability and feasibility of examining online search behaviors among women with gynecological symptoms. Despite the limited dataset, there seems to be a tendency toward heightened online search activity before patients with malignant cases visit a general practitioner. Future efforts will concentrate on expanding the dataset to facilitate the application of machine learning techniques for understanding trends in online search patterns. The goal is to develop a classification model capable of providing early indications for identifying malignant gynecological diagnoses. |
| Cohen Zion M, Gescheit I, Levy N, Yom-Tov E. Identifying sleep disorders from search engine activity: combining user-generated data with a clinically validated questionnaire. J Med Internet Res 2022 Nov 23; 24(11):e41288. PMID: 36416870 | This study explored the potential of utilizing search engine activity alongside a web-based sleep questionnaire to conduct wide-scale screening for common sleep disorders. By analyzing data from 397 participants who completed the questionnaire, the study demonstrated that diurnal patterns of individuals with sleep disorders are shifted by 2 to 3 hours compared to controls. While search engine data alone may not suffice for screening, targeted advertisements coupled with web-based tools could aid in early detection and encourage individuals to seek further assessment for sleep disorders. |
| Giat E, Yom-Tov E. Evidence from web-based dietary search patterns to the role of b12 deficiency in non-specific chronic pain: a large-scale observational study. J Med Internet Res 2018 Jan 05; 20(1):e4. PMID: 29305340 | Food-related search patterns were highly correlated with known consumption and food-related searches (ρ=.69). Awareness of B12 deficiency was associated with a higher consumption of B12-rich foods and with queries for B12 supplements. Searches for terms related to neurological disorders were correlated with searches for B12-poor foods, in contrast with control terms. Popular medicines, those having fewer indications, and those that are predominantly used to treat pain were more strongly correlated with the ability to predict neuropathic pain queries using the B12 contents of food. |
| Hochberg I, Allon R, Yom-Tov E. Assessment of the frequency of online searches for symptoms before diagnosis: analysis of archival data. J Med Internet Res 2020 Mar 06; 22(3):e15065. PMID: 32141835 | This study examined the percentage of people who search for symptoms (on Bing) before they are diagnosed with conditions that have clear physical symptoms. The study found a large variability in the percentage of people who query the internet for their symptoms before a formal medical diagnosis is made. |
| Hochberg I, Daoud D, Shehadeh N, Yom-Tov E. Can internet search engine queries be used to diagnose diabetes? Analysis of archival search data. Acta Diabetol 2019 Oct 15; 56(10):1149-54. PMID: 31093762 | Some undiagnosed diabetes patients can be detected accurately according to their symptom queries using a search engine. Such earlier diagnosis, especially in cases of type 1 diabetes, could be clinically meaningful. Search engines’ ability to serve as a population-wide screening tool could be improved using additional data provided by users. |
| Kirschenbaum MA, Birnbaum ML, Rizvi A, Muscat W, Patel L, Kane JM. Google search activity in early psychosis: a qualitative analysis of internet search query content in first episode psychosis. Early Interv Psychiatry 2020 Oct 21; 14(5):606-12. PMID: 31637869 | The study examined the Google search histories of people experiencing their initial episode of psychosis before their initial hospital admission to uncover recurring topics and subjects they were exploring during the onset of their illness. It seems that individuals in the early stages of psychosis are turning to the internet to seek information about their initial symptoms and encounters before seeking psychiatric assistance. Enhancing our comprehension of how individuals in the early phases of psychosis search online for information about their experiences could assist mental health professionals in customizing online resources. This customization could enhance access to care and potentially decrease the duration between the onset of symptoms and the initiation of appropriate treatment for psychosis. |
| Lebwohl B, Yom-Tov E. Symptoms prompting interest in celiac disease and the gluten-free diet: analysis of internet search term data. J Med Internet Res 2019 Apr 08; 21(4):e13082. PMID: 30958273 | An examination of Bing searches related to celiac disease found an increase in antecedent searches for symptoms known to be associated with celiac disease, a rise in searches for depression and anxiety, and an increase in symptoms that are associated with celiac disease but may not be reported to healthcare providers. The protean clinical manifestations of celiac disease are reflected in the diffuse nature of antecedent internet queries of those interested in celiac disease, underscoring the challenge of effective case-finding strategies. |
| Moon KC, Van Meter AR, Kirschenbaum MA, Ali A, Kane JM, Birnbaum ML. Internet search activity of young people with mood disorders who are hospitalized for suicidal thoughts and behaviors: qualitative study of google search activity. JMIR Ment Health 2021 Oct 22; 8(10):e28262. PMID: 34677139 | This study found that a significant proportion (27 out of 43, equivalent to 63%) of the participants engaged in searches related to suicide. A deeper comprehension of the searching behavior among individuals contemplating suicide can enhance strategies for outreach, evaluation, and intervention for those at risk. The utilization of search data could also offer advantages in the continuous care provided to individuals dealing with suicidal thoughts or tendencies. |
| Mueller J, Jay C, Harper S, Todd C. The role of web-based health information in help-seeking behavior prior to a diagnosis of lung cancer: a mixed-methods study. J Med Internet Res 2017 Jun 08; 19(6):e189. PMID: 28596146 | This study investigated the role of web-based information in the pathway to diagnosis for lung cancer patients. Findings indicated that although only a minority (20.4%) reported using the web before diagnosis, both patients and their next-of-kin perceived its impact across all intervals of the diagnostic pathway. The study suggests that while the current role of the web in pre-diagnosis is limited, it holds potential for reducing delays in diagnosis, especially as technology familiarity increases, and proposes the division of the diagnostic interval into two subintervals for future exploration. |
| Nitzburg G, Weber I, Yom-Tov E. Internet searches for medical symptoms before seeking information on 12-step addiction treatment programs: a web-search log analysis. J Med Internet Res 2019 Apr 04; 21(5):e10946. PMID: 31066685 | This study examined the search patterns of people prior to treatment for addiction. The results suggest that many common or non-severe medical symptoms and conditions motivate subsequent interest in addiction prevention programs. In addition to highlighting severe long-term consequences, brief interventions could be restructured to highlight how increasing substance misuse can worsen discomfort from common medical symptoms in the short term, as well as how these worsening symptoms could exacerbate social embarrassment or decrease physical attractiveness. |
| Ofran Y, Paltiel O, Pelleg D, Rowe JM, Yom-Tov E. Patterns of information-seeking for cancer on the internet: an analysis of real world data. PLoS One 2012; 7(9):e45921. PMID: 23029317 | This study investigated the searches people made on Yahoo search following their cancer diagnosis or a cancer diagnosis of their acquaintances. It shows that search data can be used to investigate medical questions on a large scale. By understanding the patterns of internet use, physicians can use this tool as a powerful partner rather than a source of distress in the care of their patients. Moreover, internet content providers need to personalize their content by taking patient search history into account, because, as the findings demonstrate, information needs change over time. |
| Paparrizos J, White RW, Horvitz E. Screening for pancreatic adenocarcinoma using signals from web search logs: feasibility study and results. J Oncol Pract 2016 Aug; 12(8):737-44. PMID: 27271506 | Signals in search logs show the possibilities of predicting a forthcoming diagnosis of pancreatic adenocarcinoma from combinations of subtle temporal signals revealed in the queries of searchers. |
| Phillips CA, Hunt A, Salvesen-Quinn M, Guerra J, Schapira MM, Bailey LC, Merchant RM. Health-related Google searches performed by parents of pediatric oncology patients. Pediatr Blood Cancer 2019 Aug 09; 66(8):e27795. PMID: 31069926 | The content found in Google searches can provide valuable insight into the concerns of parents with children diagnosed with cancer. Analyzing this content could guide us toward more inclusive strategies for educating and supporting families. The study's goal was to pinpoint the priorities and areas where parents lacked information before and after the diagnosis. Searches related to health surged in the months leading up to the child's cancer diagnosis, predominantly focusing on symptoms and logistical information. Following the cancer diagnosis, health-related searches reached their peak after about a month, and these searches included general health inquiries alongside specific cancer-related searches. |
| Sadeh-Sharvit S, Fitzsimmons-Craft EE, Taylor CB, Yom-Tov E. Predicting eating disorders from internet activity. Int J Eat Disord 2020 Sept 24; 53(9):1526-33. PMID: 32706444 | The algorithm built from internet activity reached an accuracy of 52.6% in predicting eating disorder risk/diagnostic status. The most predictive internet search history variables were the following: use of keywords related to eating disorder symptoms and websites promoting eating disorder content, participant age, median browsing events per day, and fraction of daily activity at noon. |
| Shaklai S, Gilad-Bachrach R, Yom-Tov E, Stern N. Detecting impending stroke from cognitive traits evident in internet searches: analysis of archival data. J Med Internet Res 2021 May 28; 23(5):e27084. PMID: 34047699 | The research suggested that employing algorithms utilizing online search queries could potentially identify populations at risk of stroke and predict near stroke events among those at high risk. Once it undergoes clinical validation, this algorithm holds the promise of facilitating swift preventive measures. Notably, it can be applied cost-effectively, consistently, and across a broad spectrum, with the intention of mitigating stroke events. |
| Soldaini L, Yates A, Yom-Tov E, Frieder O, Goharian N. Enhancing web search in the medical domain via query clarification. Inf Retrieval J 2015 Jul 16; 19(1-2):149-73. DOI: 10.1007/S10791-015-9258-Y | This study explored the effectiveness of bridging the gap between layperson and expert medical vocabularies through query clarification, aiming to assist users in finding authoritative and relevant medical information online. Through task-based retrieval studies and the implementation of a supervised classifier to select appropriate synonym mappings, the proposed system demonstrated improved user preference and accuracy in answering medical questions, with up to a 7% increase in correct answers. Additionally, the introduction of the classifier further enhanced the system's performance, resulting in a 12% increase in the fraction of correct answers. |
| Soldaini L, Yom-Tov E. Inferring individual attributes from search engine queries and auxiliary information. arXiv. Preprint posted online October 26, 2016 2016; :0. DOI: 10.1145/3038912.3052629 | The study introduced an algorithm designed to identify specific traits of interest in anonymous internet users, which is essential for conducting research on various human behaviors including medical conditions. By leveraging labeled examples and statistical data about the population, the algorithm can accurately assign labels to unseen examples, facilitating research in domains where direct identification is challenging due to privacy concerns. The algorithm's applications in the medical domain demonstrate its effectiveness in identifying potential cancer patients based on search patterns and in predicting disease distributions within a population, offering valuable insights for early disease screening and epidemiological studies. |
| Tang H, Ng JH. Googling for a diagnosis--use of Google as a diagnostic aid: internet based study. BMJ 2006 Dec 02; 333(7579):1143-5. PMID: 17098763 | Google searches revealed the correct diagnosis in 15 (58%, 95% confidence interval 38% to 77%) cases. As internet access becomes more readily available in outpatient clinics and hospital wards, the web is rapidly becoming an important clinical tool for doctors. The use of web-based searching may help doctors diagnose difficult cases. |
| White R, Horvitz E. From web search to healthcare utilization: privacy-sensitive studies from mobile data. J Am Med Inform Assoc 2013 Jan 01; 20(1):61-8. PMID: 22661560 | Aiming to understand the relationship between health information- seeking behavior and engagement with healthcare professionals, this study conducted a privacy-conscious analysis of geo-tagged data from mobile devices. By analyzing anonymized logs of mobile interactions focusing on search queries and distances to medical care centers, the study investigated the sequence of health-related searches leading to observed healthcare utilization indicators. Results revealed that the duration between symptom searches and evidence of healthcare utilization varies depending on symptom severity. Statistical models were developed to predict forthcoming healthcare utilization that achieved predictive accuracies ranging from 65% to 90%. The study emphasizes the importance of privacy- sensitive analysis in generating insights into health information seeking and healthcare engagement, highlighting the potential of large-scale mobile device studies in understanding individuals’ pathways to professional medical care. |
| White RW, Horvitz E. Evaluation of the feasibility of screening patients for early signs of lung carcinoma in web search logs. JAMA Oncol 2017 Mar 01; 3(3):398-401. PMID: 27832243 | A statistical classifier accurately identified web searchers who later input queries that provided evidence of a recent clinical diagnosis of lung carcinoma. The methods can help identify people at highest risk up to a year in advance of the inferred diagnosis time and identify new risk factors (e.g., house, age, air travel patterns) expressed as evidence in people’s search activity and geographic location. |
| White RW, Horvitz E. From health search to healthcare: explorations of intention and utilization via query logs and user surveys. J Am Med Inform Assoc 2014; 21(1):49-55. PMID: 23666794 | This study aimed to understand the relationship between online health-seeking behaviors and in-world healthcare utilization (HU) by analyzing data from online searches and surveys. By examining search logs and survey responses, the study provides insights into how users transition from online health information seeking to utilizing professional healthcare services. Results suggest a strong correlation between search behavior and healthcare utilization, offering the potential for inferring HU from long-term search  patterns without tracking physical location, which could enhance models of user interests and preferences. |
| Yom-Tov E, Cherlow Y. Ethical challenges and opportunities associated with the ability to perform medical screening from interactions with search engines: viewpoint. J Med Internet Res 2020 Sept 16; 22(9):e21922. PMID: 32936082 | The study explored the potential of utilizing search engine logs for screening various medical conditions, offering opportunities for earlier diagnosis and equitable healthcare access but raising ethical concerns regarding privacy and autonomy. It discusses different approaches for providing screening information, from displaying notices to modifying search results and using advertisements, each with its advantages and disadvantages in terms of privacy, autonomy, and effectiveness. The study emphasizes the need for careful consideration of the ethical implications and suggests  solutions that balance the benefits of online screening services with the challenges they pose. |
| Yom-Tov E, Navar I, Fraenkel E, Berry JD. Identifying amyotrophic lateral sclerosis through interactions with an internet search engine. Muscle Nerve 2024 Jan 25; 69(1):40-7. PMID: 37877320 | The study investigated the feasibility of using internet search engine interactions to identify individuals with amyotrophic lateral sclerosis (ALS), with the goal of reducing the time from symptom onset to diagnosis. Through analysis of search engine query data from ALS patients and control groups, the study developed a model that distinguishes between ALS cases and controls with promising accuracy (AUC of 0.81). Prospective validation supports the potential of search engine interactions as a screening tool for ALS, although distinguishing ALS from disease mimics remains challenging. These findings underscore the need for further research to explore the role of search engine data in ALS diagnosis and in reducing diagnostic delays. |
| Yom-Tov E. Screening for cancer using a learning internet advertising system. ACM Trans Comput Healthcare 2020 Mar 11; 1(2):1-13. DOI: 10.1145/3373720 | The study demonstrated the effectiveness of using online advertising systems such as Bing and Google ads in identifying individuals who may have symptoms consistent with suspected cancer. By providing clinically verified questionnaires and analyzing responses, a classifier trained on past Bing queries achieved a predictive accuracy of 0.64 for suspected cancer. Furthermore, leveraging questionnaire responses within Google's advertisement system enabled the identification of individuals likely to have suspected cancer, highlighting the potential of modern advertising platforms to aid in the early detection of serious medical conditions. |
| Yom-Tov E, White RW, Horvitz E. Seeking insights about cycling mood disorders via anonymized search logs. J Med Internet Res 2014 Feb 25; 16(2):e65. PMID: 24568936 | This study examined Bing searches of people with cycling mood disorders. |
| Youngmann B, Allerhand L, Paltiel O, Yom-Tov E, Arkadir D. A machine learning algorithm successfully screens for Parkinson's in web users. Ann Clin Transl Neurol 2019 Dec 12; 6(12):2503-9. PMID: 31714022 | The study aimed to create and assess a novel web-based classifier for Parkinson's disease screening using search engine user data. Applying the classifier to a large cohort identified 1.2% of users over 40 years old as screening positive for Parkinson's, with higher rates in at-risk groups. Longitudinal analysis revealed faster disease progression in individuals classified as positive, highlighting the potential of web-based screening but also raising ethical concerns. |
| Youngmann B, Yom-Tov E. Anxiety and information seeking: evidence from large-scale mouse tracking. In: Proceedings of the 2018 World Wide Web Conference. 2018. Presented at: WWW '18; April 23-27, 2018; Lyon, France. p. 753-62. DOI: 10.1145/3178876.3186156 | The study revealed that individuals exhibit distinct information- seeking behaviors on search engines depending on their level of anxiety and that this is particularly evident in searches for medical symptoms with potentially life-threatening implications. By analyzing mouse tracking data and other user interactions, a model is developed to predict user anxiety levels, achieving significant correlation with the severity of symptoms searched. The findings underscore the importance of incorporating user anxiety information to accurately measure search engine performance. This is particularly crucial in delivering critical medical information and suicide prevention resources effectively. |
| Youngmann B, Yom-Tov E. Intimate partner violence as reflected in internet search data. Soc Sci Comput Rev 2022 Apr 12; 41(5):1546-61. DOI: 10.1177/08944393221084074 | Queries from Bing search engine data of more than 50,000 US-based individuals suffering from IPV were extracted and analyzed. Approximately half of the users begin to search for IPV following an acute event (physical violence or abuse), and 20% of users actively hide their interest in IPV. The topics of interest to people who experience IPV include the effects of IPV, help-seeking, and methods to escape from IPV. Early cues of IPV may be difficult to detect within search queries, and in the late stage in which many IPV users are identified, interventions such as ads to guide people to safely exit violent situations could be beneficial. |
| Zaman A, Acharyya R, Kautz H, Silenzio V. Detecting low self-esteem in youths from web search data. In: Proceedings of the 2019 Conference on World Wide Web. 2019. Presented at: WWW '19; May 13-17, 2019; San Francisco, CA. p. 2270-80. DOI: 10.1145/3308558.3313557 | This study addressed the void in examining indicators of low self- esteem, a condition intricately linked to a cycle involving depression and anxiety, at an individual level through the analysis of Google search history data. The research focuses on college students, a demographic susceptible to experiencing depression, anxiety, and low self-esteem. They are asked to complete a mental health assessment survey and to provide access to their individual search history. Textual analysis of the search logs reveals prominent patterns capable of identifying individuals currently experiencing low self-esteem. Notably, participants with low self-esteem display fewer searches related to family, friends, and financial aspects. Furthermore, observable differences in the distribution of search categories over time distinguish them from individuals exhibiting moderate to high self-esteem. Leveraging these distinctive markers, the study developed a probabilistic classifier capable of detecting low self-esteem conditions based on search history, achieving an average F1 score of 0.86. |
| Zaman A, Kautz H, Silenzio V, Hoque ME, Nichols-Hadeed C, Cerulli C. Discovering intimate partner violence from web search history. Smart Health 2021 Mar; 19:100161. DOI: 10.1016/j.smhl.2020.100161 | This paper proposed an adaptable, lightweight, and widely applicable screening method, validated through authentic data obtained from self-assessment surveys. This technique aims to identify potential indicators of intimate partner violence (IPV) by examining individual Google search histories. Preliminary analysis reveals discernible temporal, textual, and contextual differences in search behaviors between individuals who have or have not encountered IPV. Leveraging these distinctive patterns, a model capable of identifying violence within intimate relationships was constructed, achieving an F1 score of 0.80. Although these findings are preliminary, it is anticipated that this research will stimulate the AI community to address this critical public health issue. Additionally, this study illustrates a clear schematic depicting the processes of consent, data download, and linkage. |
| Zaman, Anis. University of Rochester Libraries. Combining traditional and non-traditional data stream for understanding mental health [accessed 2025-01-08]. http://hdl.handle.net/1802/36286 | This dissertation explored daily online behaviors via Google Search and YouTube platforms to create predictive models for various mental health conditions and introduced a cloud-based framework merging verified mental health indicators with daily online activities, enabling model construction for diverse mental health issues. During a 2-month study involving a college population, Google search logs revealed strong signals identifying individuals with low self-esteem. This mental health assessment framework is cost-effective, time-saving, and scalable, potentially applicable in real-world clinical settings. It enables healthcare providers to non- invasively understand patients’ anxiety disorders at any given moment. |
| Zaman A, Zhang B, Silenzio V, Hoque E, Kautz H. Individual-level anxiety detection and prediction from longitudinal YouTube and Google search engagement logs. arXiv. Preprint posted online July 1, 2020 2020; :0. DOI: 10.48550/arxiv.2007.00613 | The paper introduced an innovative method aimed at identifying individuals experiencing anxiety and assessing the severity of their anxiety levels by analyzing their personal online activity histories obtained from popular platforms such as YouTube and Google Search. These platforms serve millions of users daily. The study conducted a longitudinal analysis, gathering multiple sets of anonymized YouTube and Google Search logs from volunteers, alongside clinically validated anxiety assessment scores. Subsequently, the researchers devised distinct features capturing the temporal and contextual aspects of online behaviors. Utilizing these features, the team trained models capable of (i) detecting individuals with anxiety disorder, achieving an average F1 score of 0.83 ± 0.09, and (ii) estimating anxiety levels by predicting the widely accepted Generalized Anxiety Disorder 7-item scores (ranging from 0 to 21) with a mean square error of 1.87 ± 0.15, leveraging ubiquitous individual-level online engagement data. The proposed framework for assessing anxiety proves to be cost-effective, time-efficient, and scalable, and it holds promise for real-world implementation in clinical settings. This approach enables healthcare providers and therapists to non-invasively understand patients’ anxiety disorders at any given moment, providing valuable insights for personalized care. |
| Zaman A, Zhang B, Silenzio V, Kautz H, Hoque E. The relationships of deteriorating depression and anxiety with longitudinal behavioral changes in Google and YouTube use during COVID-19: observational study. JMIR Ment Health 2020 Nov 23; 7(11):e24012. PMID: 33180743 | This study investigated the relationship between changes in Google search and YouTube engagement behaviors and the exacerbation of depression and anxiety levels among college students during the COVID-19 pandemic. Through longitudinal data collection and correlation analysis, the study identified significant associations between deteriorating mental health profiles and shifts in online behavior, suggesting the potential utility of these behavioral changes as predictive indicators of mental health conditions. |
| Zhang B, Zaman A, Acharyya R, Hoque E, Silenzio V, Kautz H. Detecting individuals with depressive disorder from personal Google search and YouTube history logs. arXiv. Preprint posted online October 28, 2020 2020; :0. DOI: 10.48550/arxiv.2010.15670 | This study proposed a personalized framework utilizing Google Search and YouTube engagement logs to detect individuals with depressive disorder, offering a more accessible and timely screening method compared to traditional in-person interviews. |
