# Supplementary material for: Harnessing Internet Search Data as a Potential Tool for Medical Diagnosis: Literature Review
Source: JMIR Ment Health. 2025 Feb 11;12:e63149. doi: 10.2196/63149 (PMC11862766; doi:10.2196/63149)
Supplement: Multimedia Appendix 5 [file mental_v12i1e63149_app5.docx]

Appendix 5: Tools Developed to Assist Researchers in the Use of Search Data

| **Tool Name** | **Function** |
| --- | --- |
| gTAP Web App | This web app allows participants to download their data without sharing personal Google account credentials [39]. |
| LIWC | This is a text analysis software package that can differentiate linguistic attributes in search logs [36]. Previous researchers have performed psycholinguistic analysis on text data to uncover signals of abuse (emotional, sexual, or physical) and domestic violence using the LIWC [36]. |
| Google Natural Language AI (NLP API) | The Google NLP API assures removal of personally identifying information [48]. The API scans for personal identifiers such as names, addresses, and phone numbers that can potentially identify individual study participants, and this information is automatically removed from the search history data before it is transferred to the research team and saved as research data [48]. |
| CrowdTangle | This is a tool from Meta to help follow, analyze, and report what is happening across social media [7]. It is the most effective transparency tool in the history of social media [15]. |
| Latent Dirichlet Allocation (LDA) | LDA produces clusters of words that occur in the same context across Facebook posts, yielding semantically coherent topics [15]. It is appropriate for the highly non- normal frequency distributions observed in language use [15]. |
| Differential Language Analysis Toolkit (DLATK) | The DLATK determines the relative frequency with which users used words (unigrams) and two-word phrases (bigrams) [59]. It can also retain variables and phrases [59]. |
| GPT-4 AI Chatbot GPT-4 (OpenAI) | LLMs can convincingly solve difficult diagnostic cases, pass licensing examinations, and communicate empathetically with patients, suggesting that they have an emergent understanding of clinical reasoning [60]. This diagnostic study assessed the ability of the AI chatbot GPT-4 (OpenAI) to appropriately perform probabilistic reasoning by comparing its performance with a large survey of human clinicians [60]. |
